# Supplementary figures and images for: A cytoplasmic form of EHMT1N methylates viral proteins to enable inclusion body maturation and efficient viral replication
Source: PLoS Biol. 2024 Nov 7;22(11):e3002871. doi: 10.1371/journal.pbio.3002871 (PMC11575796; doi:10.1371/journal.pbio.3002871)

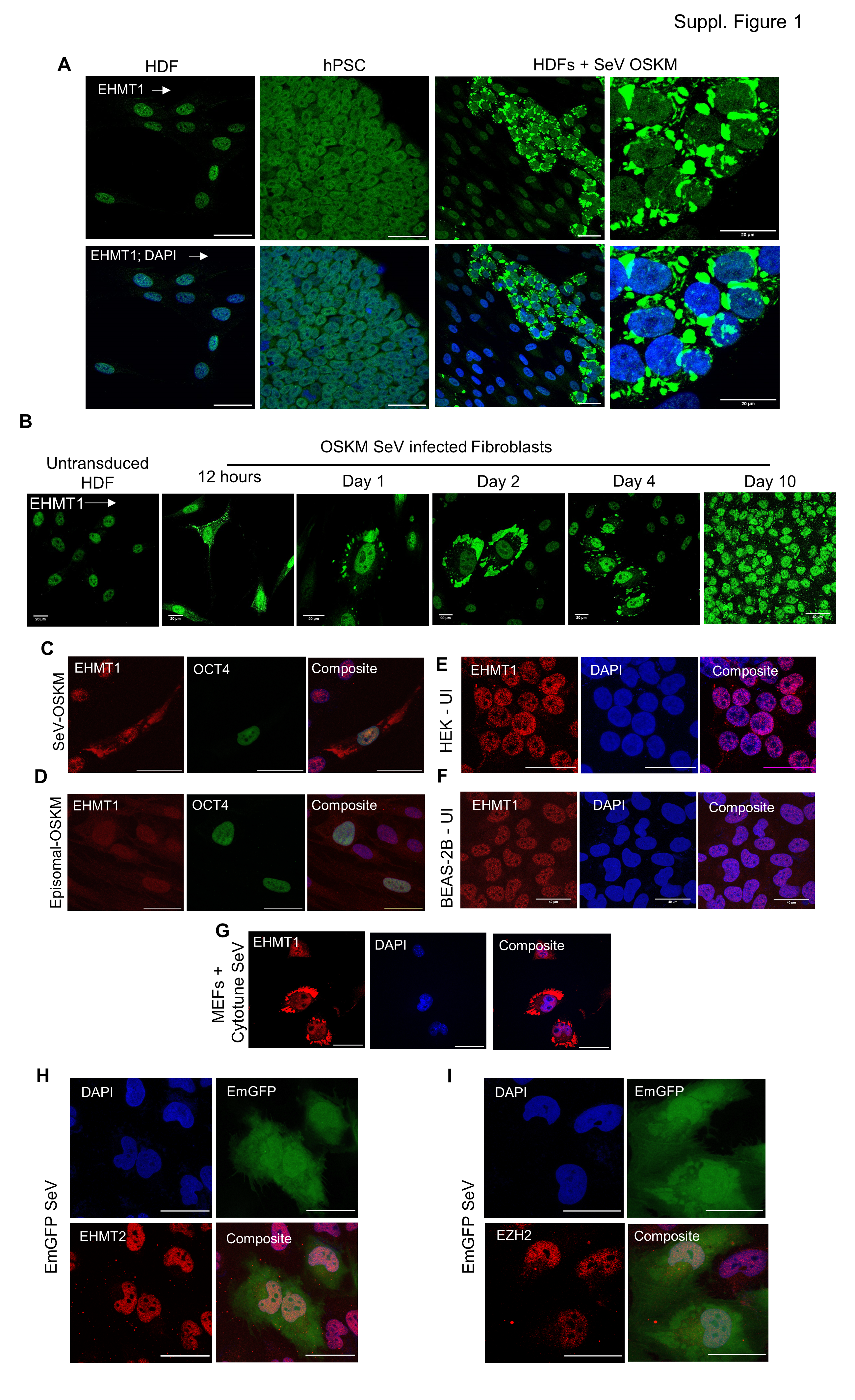

Supplement: S1 Fig — Confocal microscopic images of (A) various stages of fibroblasts undergoing reprogramming induced by the ectopic expression of OSKM delivered via SeV, immunolabelled with EHMT1 (green). (B) Fibroblasts transduced with OSKM SeV, immunolabelled with EHMT1 (green), at indicated time points. (C) Fibroblasts transduced with OSKM via SeV, immunolabelled for EHMT1 (red) and Oct4 (green). (D) Fibroblasts transfected with episomal plasmids expressing OSKM, immunolabelled with EHMT1 (red) and Oct4 (green). (E) Uninfected HEK immunolabelled with EHMT1 (red), (F) uninfected BEAS-2B immunolabelled with EHMT1 (red). (G) Mouse embryonic fibroblasts (MEFs) infected with SeV, immunolabelled with EHMT1 (red). (H, I) BEAS-2B infected with EmGFP SeV (green) immunolabelled with (H) EHMT2 (red) and (I) Ezh2 (red). Composite of images are with DAPI (blue) stained nuclei. Scale bar, 40 μm. Raw confocal microscopic images are deposited on BioImage Archive (Accession id: S-BIAD1362). (TIF) [file pbio.3002871.s001.TIF]

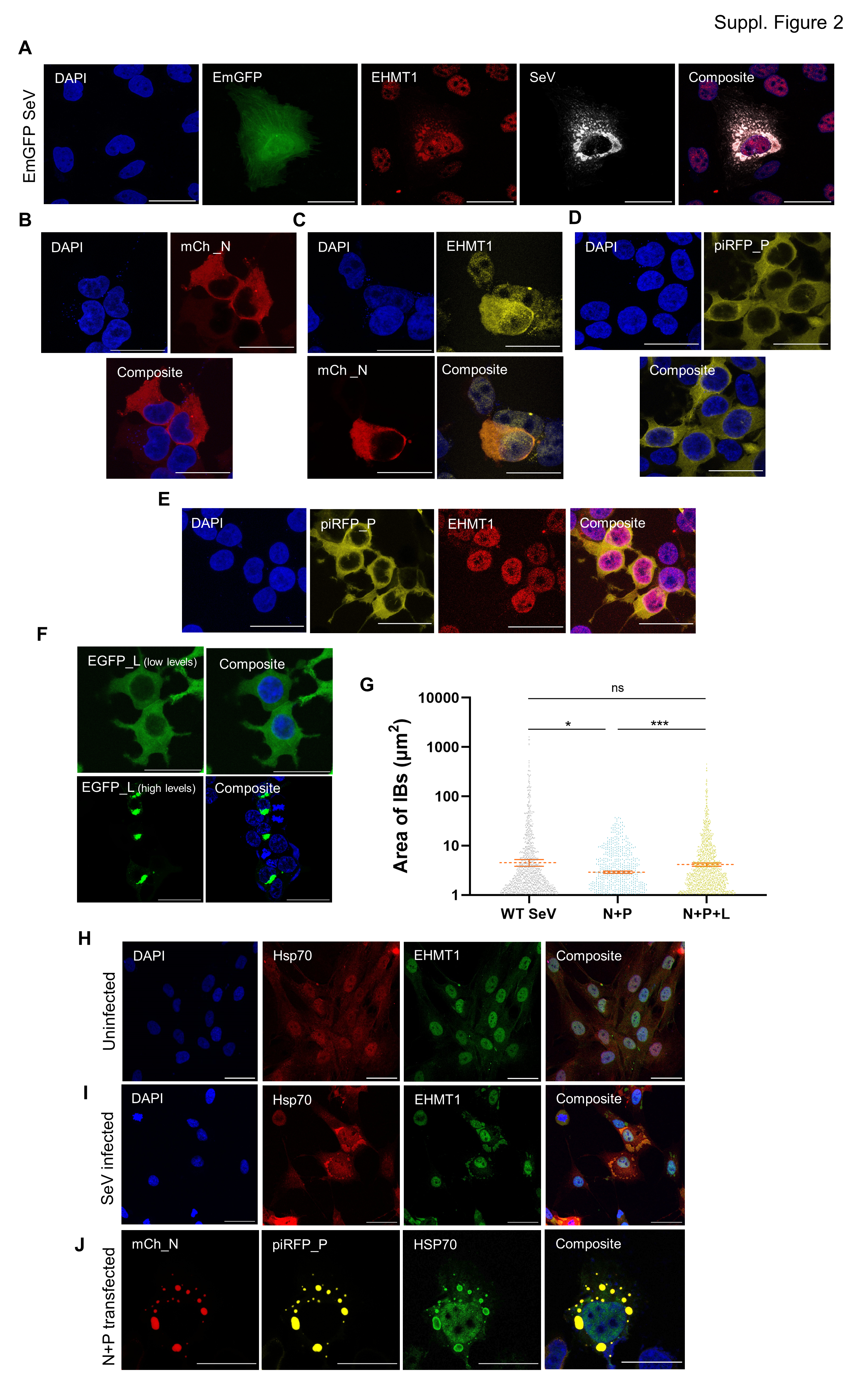

Supplement: S2 Fig — Confocal microscopic images of (A) BEAS-2B infected with EmGFP SeV (green) immunolabelled with EHMT1 (red) and SeV (grey). HEK transfected with (B) mCh_N (red) and (D) piRFP_P (yellow), (C) HEK transfected with mCh_N (red) and immunolabelled with EHMT1 (yellow), (D) HEK transfected with piRFP_P (yellow) and immunolabelled with EHMT1 (red). (F) HEK transfected with EGFP_L (green). (G) Graph representing the overall area of all IBs formed in cells across infection and co-transfection conditions. (n = 3 replicates, unpaired t test with Welch’s correction between the indicated groups, p-value: 0.1234 (ns), 0.0332 (*), 0.0002 (***)) (H) Fibroblast uninfected and (I) infected with SeV co-immunolabelled with Hsp70 (red) and EHMT1 (green). (J) HEKs co-transfected with mCh_N (red) and piRFP_P (yellow) in a 1:1 ratio, immunolabelled with Hsp70 (green). Composite of all images are with DAPI (blue) stained nuclei. Scale bar, 40 μm. Source data are provided as S1 Data. Raw confocal microscopic images are deposited on BioImage Archive (Accession id: S-BIAD1362). (TIF) [file pbio.3002871.s002.TIF]

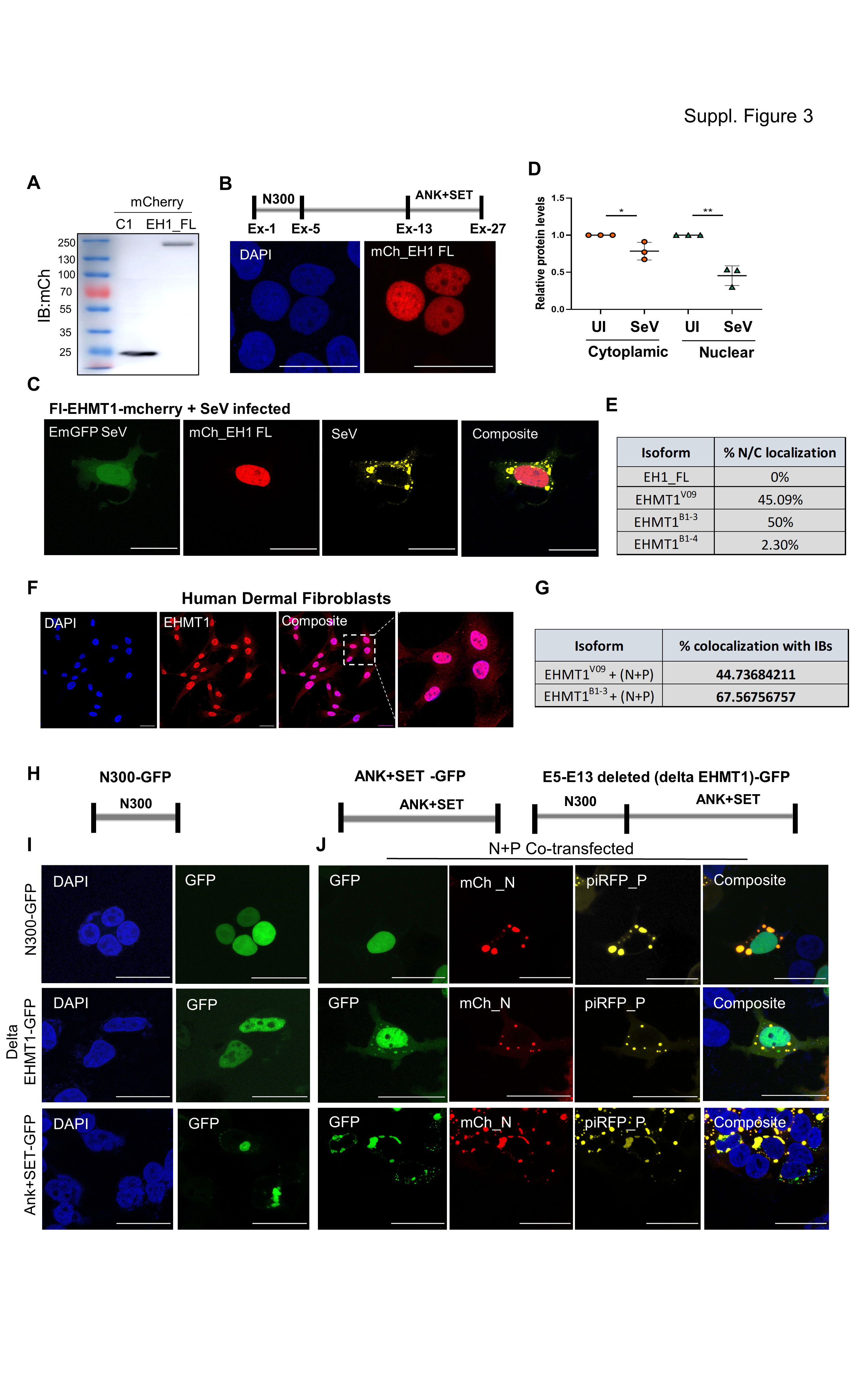

Supplement: S3 Fig — (A) Whole cell lysate of HEKs transfected with mCh_C1 or mCh_EH1_FL, western blotted and probed with mCherry. Confocal microscopic images of HEK transfected with (B) mCh_EH1_FL (red), (C) mCh_EH1_FL (red), infected with EmGFP SeV (green) and immunolabelled with SeV (yellow). Nuclei stained blue with DAPI. (D) Graph representing relative protein levels of EHMT1 quantified from western blots of nuclear and cytoplasmic fractions at 24 h p.i. EHMT1 bands from SeV infected lanes were normalised against uninfected lanes (n ≥ 3 replicates, one-way ANOVA, p-value: 0.0332 (*), 0.0021 (**)). (E, G) HEK were transfected with either mCh_EH1_FL, EHMT1B1-3_Egfp, EHMT1B1-4_Egfp, or EHMT1V09_Egfp. Table representing (E) the percentage of transfected cells demonstrating nucleo-cytoplasmic signal, (G) co-transfected with mCh_N and piRFP_P; table representing % triple transfected cells with colocalization of various forms of EHMT1 into IBs. (F) Confocal microscopic images of fibroblasts immunolabelled with EHMT1 (red) acquired by adjusting the threshold corrected for background with a secondary control. (H) Schematic representation of EHMT1 truncated sequences cloned into EGFP reporter plasmid. (I) Confocal microscopic images of HEK transfected individually with N300_EGFP, Delta EHMT1_EGFP and Ank+SET_EGFP. (J) Confocal microscopic images of HEK transfected with N300-GFP, Delta EHMT1_EGFP and Ank+SET_EGFP, co-transfected with mCh_N + piRFP_P, composite images are with DAPI (blue) stained nuclei. Scale bar, 40 μm. Source data are provided as S1 Data. Raw confocal microscopic images are deposited on BioImage Archive (Accession id: S-BIAD1362). (TIF) [file pbio.3002871.s003.TIF]

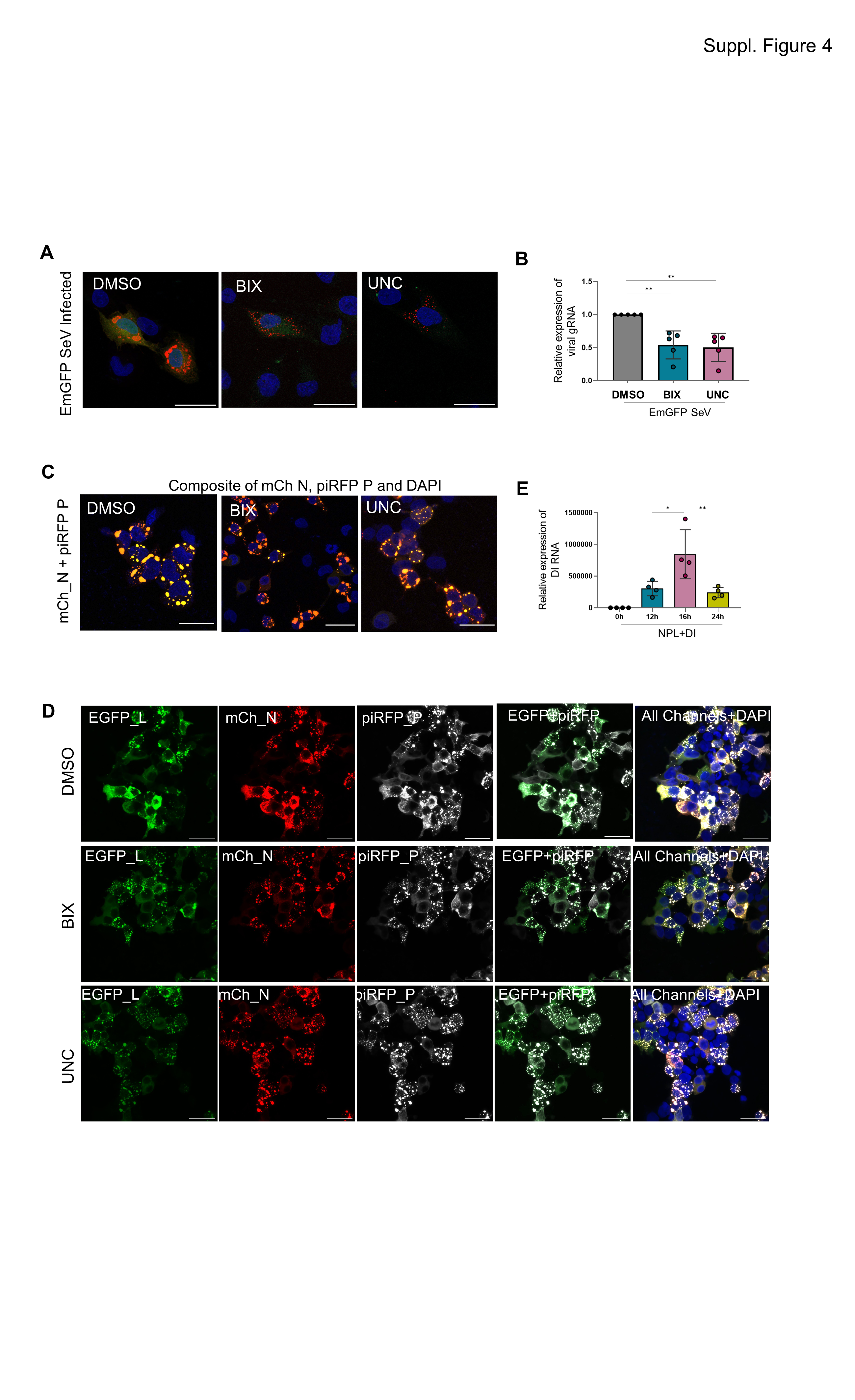

Supplement: S4 Fig — (A) Confocal microscopic composite images of EmGFP SeV (green) infected BEAS-2B cells, simultaneously treated with DMSO, 3 μm BIX or 3 μm UNC, immunolabelled with SeV ab (red), marking the IBs. (B) Graph plotted for the relative expression of SeV gRNA assessed by qRT-PCR with the Ct values normalised against GAPDH (n = 4 replicates, one-way ANOVA, **, p < 0.005). (C) Confocal microscopic composite images of HEK transfected with mCh_N (red) and piRFP_P (yellow), treated with DMSO, 3 μm BIX or 3 μm UNC. (D) Confocal microscopic images of HEK co-transfected with EGFP_L (green), mCh_N (red), and piRFP_P (grey), treated with DMSO, 3 μm BIX or 3 μm UNC. Composite images are with DAPI (blue) stained nuclei. Scale bar, 40 μm. (E) Graph representing the relative expression of SeV DI RNA at indicated time points post transfection, as assessed by qRT-PCR with Ct values normalised against GAPDH. (n = 4 replicates, one-way ANOVA, p < 0.005 (**), p < 0.05 (*)) Source data are provided as S1 Data. Raw confocal microscopic images are deposited on BioImage Archive (Accession id: S-BIAD1362). (TIF) [file pbio.3002871.s004.TIF]

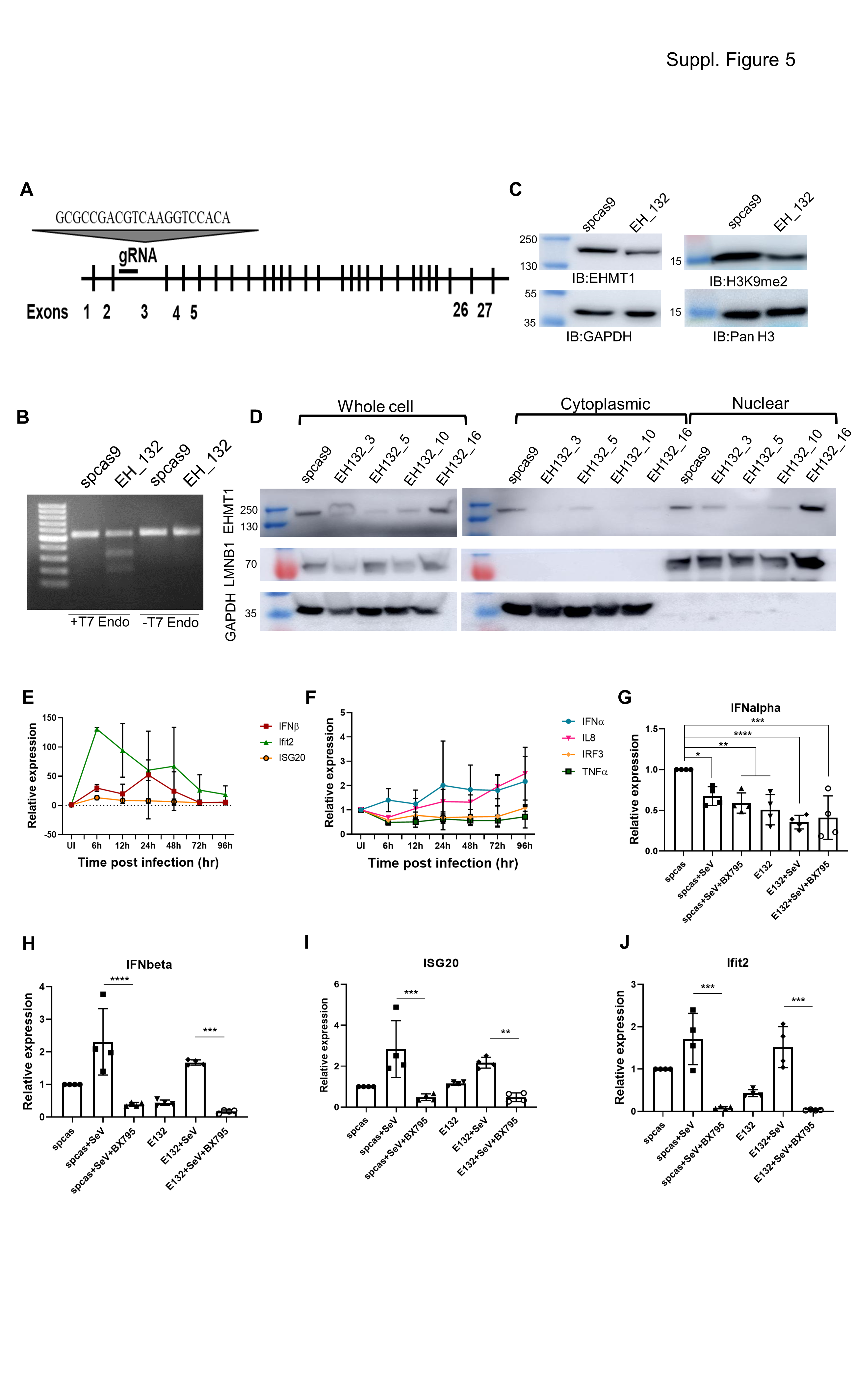

Supplement: S5 Fig — (A) Schematic and sequence of EH_132 guide RNA targeting exon 3 of EHMT1 via CRISPR/Cas9 method of gene editing. (B) Agarose gel electrophoresis of the products from T7 endonuclease assay demonstrating gene editing as seen by digestion into 3 products in EH_132 mutated sample. (C) Western blotting of the SpCas9 control and EH_132 lysates immunoblotted with EHMT1, GAPDH, H3K9me2, and panH3. (D) Western blotting of the whole cell, nuclear and cytoplasmic fractions of single cell clones of EH_132, probed with EHMT1, LaminB1, and GAPDH. (E, F) Graphs representing relative expression of Type 1 IFN and stimulated genes over the time course of infection with WT SeV, as assessed by qRT-PCR. (G–J) HEK were transfected with SpCas9 or E132 (EH_132), infected with WT SeV and simultaneously treated with BX795; qRT-PCR was performed to assess the relative expression of (G) IFNα, (H) IFNβ, (I) ISG20, and (J) Ifit2 across the indicated conditions. (n = 4 replicates, one-way ANOVA, p < 0.05 (*), p < 0.005 (**), 0.0002 (***), <0.0001 (****)) Source data are provided as S1 Data. (TIF) [file pbio.3002871.s005.TIF]

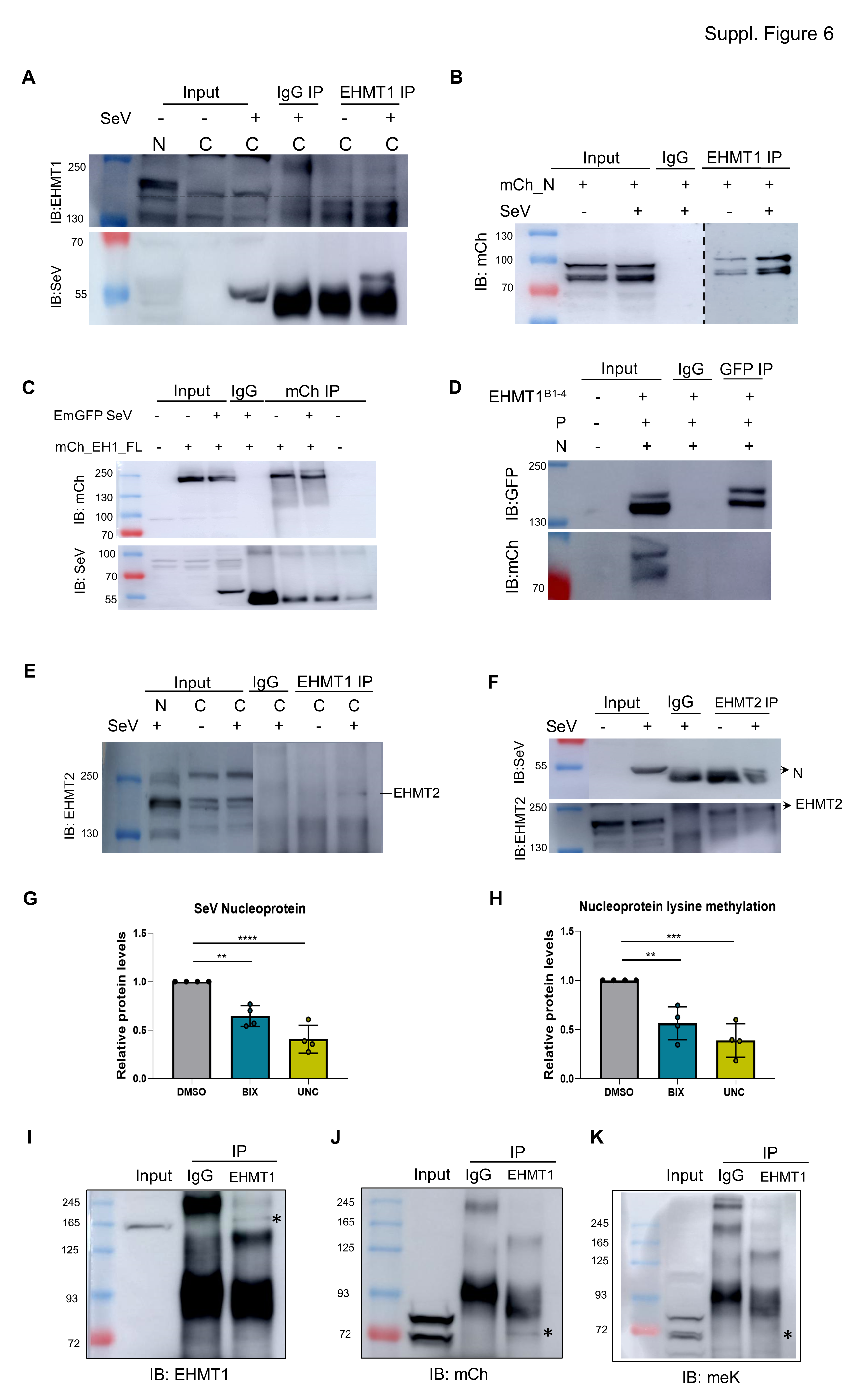

Supplement: S6 Fig — (A) EHMT1 IP from the cytoplasmic fraction of SeV infected or uninfected cells, elute western blotted and probed with EHMT1 and SeV. Dotted line in black indicates the nucleo-cytoplasmic form of EHMT1. (B) EHMT1 IP from the cytoplasmic fraction of cells transfected with mCh_N, infected with SeV, elute western blotted and probed with mCherry. (C) mCherry IP from whole cell lysate of HEK transfected with mCh_EH1_FL and infected with EmGFP SeV; the elute was western blotted and probed with mCherry and SeV, depicting no association between mCh_EHMT1_FL and SeV proteins. (D) GFP IP from cells triple transfected with EHMT1B1-4_EGFP, mCh_N and piRFP_P; elute was western blotted and probed with GFP and mCherry. (E) EHMT1 IP from the cytoplasmic fractions of uninfected or SeV infected cells; elute western blotted and probed with EHMT2, depicting an interaction between EHMT1 and EHMT2. (F) EHMT2 IP from the cytoplasmic fraction of SeV infected and uninfected cells, elute western blotted and probed with SeV and EHMT2. (G, H) Graphs representing quantification of the (G) SeV nucleoprotein and (H) meK levels from western blots (Fig 6I). For (G), protein levels were normalised against GAPDH and for (H), protein levels were normalised against the respective nucleoprotein bands. (n = 4 replicates, one-way ANOVA, **, p < 0.005, 0.0002 (***), <0.0001 (****)) (I–K) HEK were co-transfected with mCh_N and piRFP_P, EHMT1N/C was immunoprecipitated from the cytoplasmic fraction 24 h post transfection. IP elute was western blotted and probed with (I) EHMT1, (J) mCherry, and (K) meK. Source data are provided as S1 Data. (TIF) [file pbio.3002871.s006.TIF]

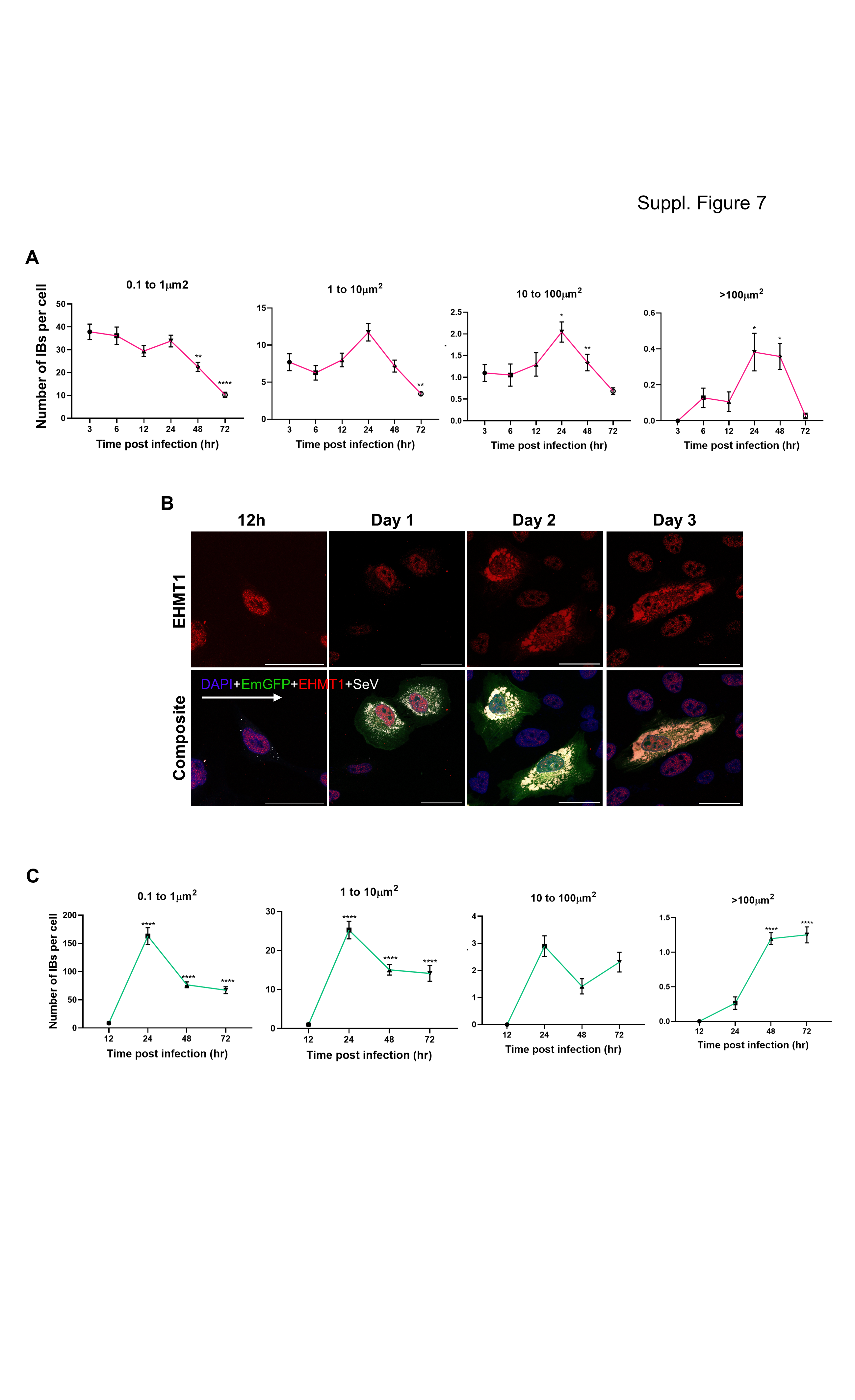

Supplement: S7 Fig — (A) Graphs representing the mean number of IBs (y-axis) in each subpopulation for WT SeV (pink line) infected cells. Data are ±SEM. (n > 25 cells, Brown–Forsythe and Welch ANOVA tests) p-value: 0.1234 (ns), 0.0332 (*), 0.0021 (**), 0.0002 (***), <0.0001 (****). (B) Confocal microscopic images of BEAS-2B cells infected with EmGFP SeV (green) immunolabelled with EHMT1 (red) and SeV (grey) at indicated time points postinfection, composite images of all channels are with DAPI (blue) stained nuclei. Scale bar, 40μm. (C) Graphs representing the mean number of IBs (y-axis) in each subpopulation for EmGFP SeV (green line) infected cells. Data are ±SEM. (n > 25 cells, Brown–Forsythe and Welch ANOVA tests) p-value: 0.1234 (ns), 0.0332 (*), 0.0021 (**), 0.0002 (***), <0.0001 (****). Source data are provided as S1 Data. Raw confocal microscopic images are deposited on BioImage Archive (Accession id: S-BIAD1362). (TIF) [file pbio.3002871.s007.TIF]

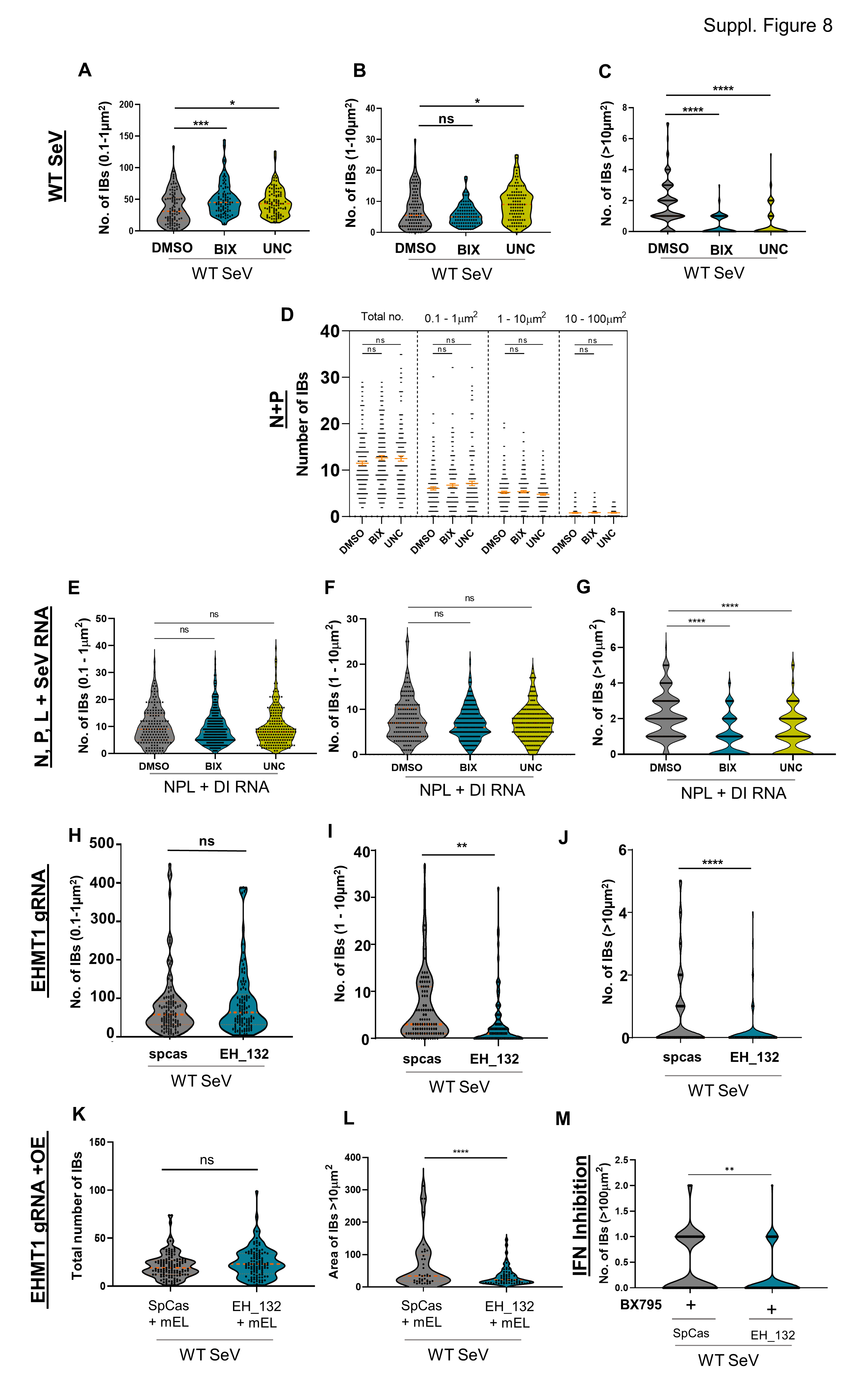

Supplement: S8 Fig — (A–N) Graphs representing the number and area of IBs as quantified from confocal microscopic images using the ImageJ analysis tool. (A–C) BEAS-2B cells were infected with WT SeV and treated with 3 μm of BIX/UNC simultaneously. The cells were fixed and immunolabelled with SeV antibody 16 h p.i. Graphs representing number of IBs in (A) 0.1–1 μm2 category, (B) 1–10 μm2 category, and (C) >10 μm2 category. (N = 3 replicates, n > 100 cells, ordinary one-way ANOVA, p-value: 0.1234 (ns), 0.0332 (*), 0.0002 (***), <0.0001 (****)) (D) Graph representing the total number of IBs of various subpopulations in N+P co-transfected cells, treated with 3 μm of BIX/UNC. (N = 5 replicates, n > 300 cells, ordinary one-way ANOVA, 0.1234 (ns)) (E–G) HEK were co-transfected with mCh_N, piRFP_P, and EGFP_L, followed by transfection with SeV DI RNA 12 h post transfection of the plasmids, either untreated or treated with 3 μm of BIX/UNC. Graphs representing number of IBs in (E) 0.1–1 μm2 category, (F) 1–10 μm2 category, and (G) >10 μm2 category. (N = 2 replicates, n > 200 cells, ordinary one-way ANOVA, 0.1234 (ns), <0.0001 (****)). (H–J) HEK were transfected with empty SpCas9 or EH_132 plasmid to deplete the levels of EHMT1 and infected with WT SeV, cells were immunolabelled with SeV 16 h p.i. Graphs representing number of IBs in (H) 0.1–1 μm2 category, (I) 1–10 μm2 category, (J) >10 μm2 category. (N = 3 replicates, n > 100 cells, unpaired t test) p-value: 0.1234 (ns), 0.0021 (**), <0.0001 (****)). (K, L) HEK293 were transfected with SpCas9 empty plasmid or EH_132, and mCh_EH1_FL (mEL); the cells were then infected with WT SeV and immunolabelled for SeV 16 h p.i. (K) Graph representing the total number of IBs and (L) area of IBs >10 μm2. (N = 2 replicates, n > 100 cells, unpaired t test) p-value: 0.1234 (ns), <0.0001 (****)) (M) HEKs were transfected with SpCas9 or EH_132, infected with WT SeV and simultaneously treated with BX795. Graph representing the number of IBs >100 μm2. (N = 3 replicat [file pbio.3002871.s008.TIF]

3A

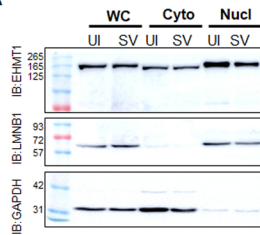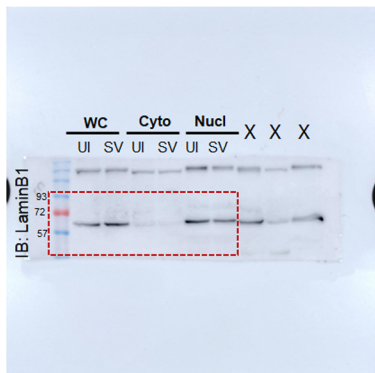

3B

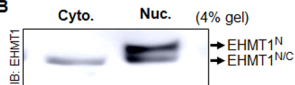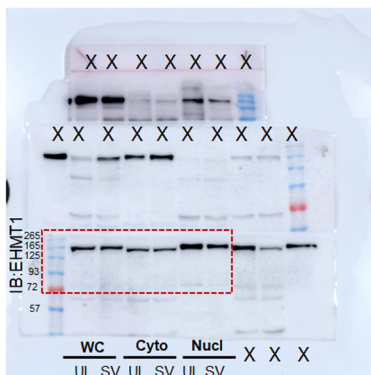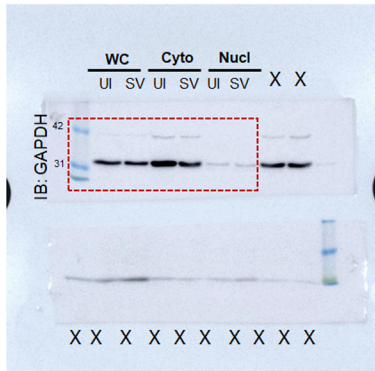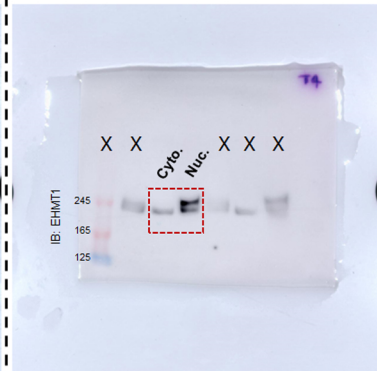

6A

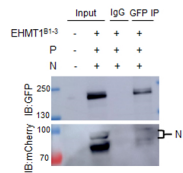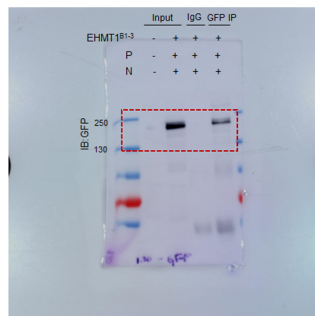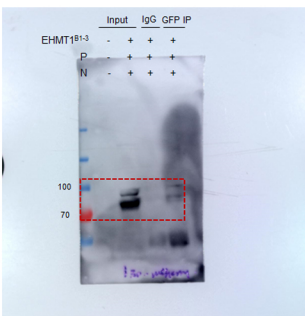

6B

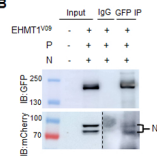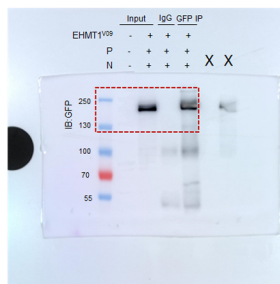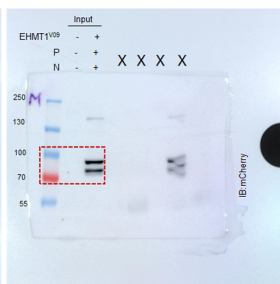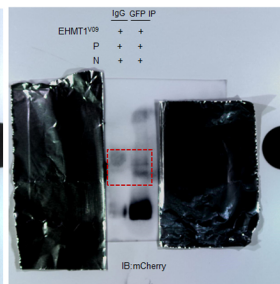

6C

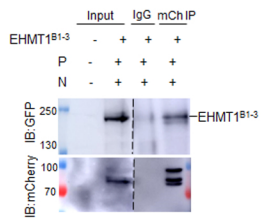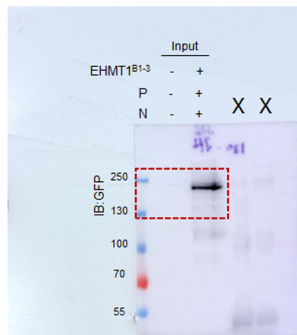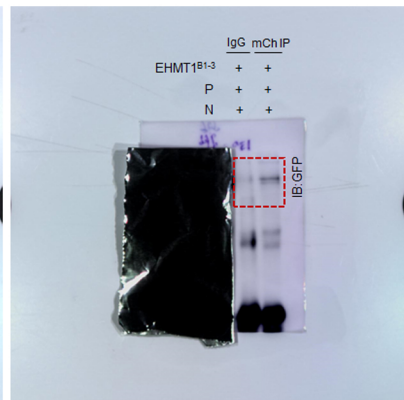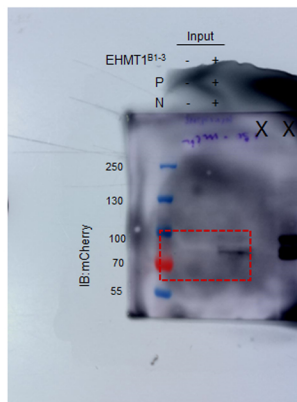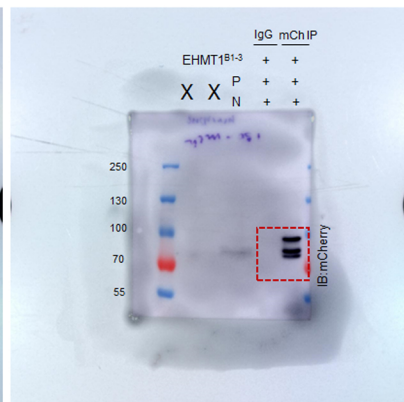

6D

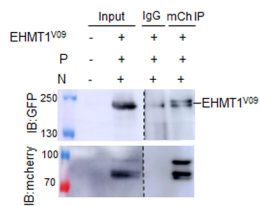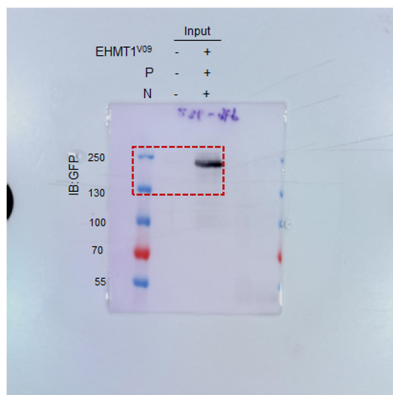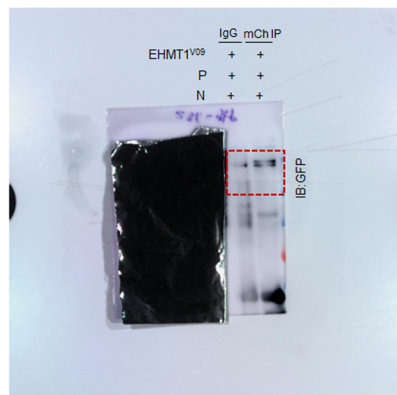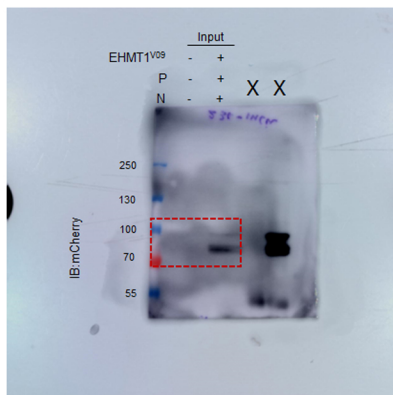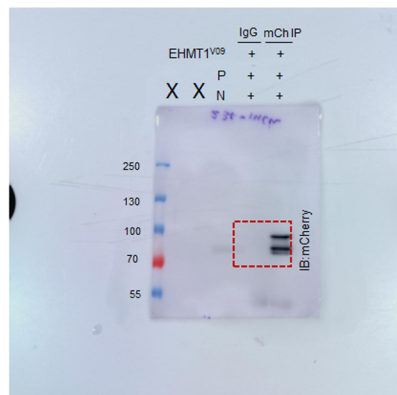

6E

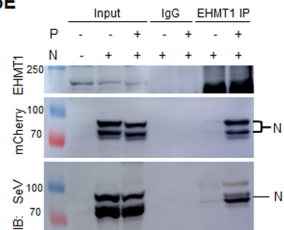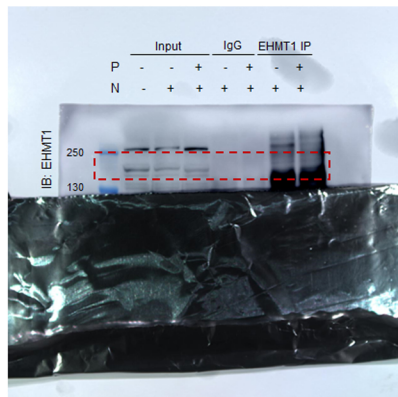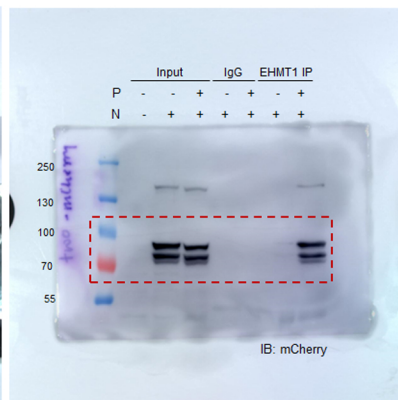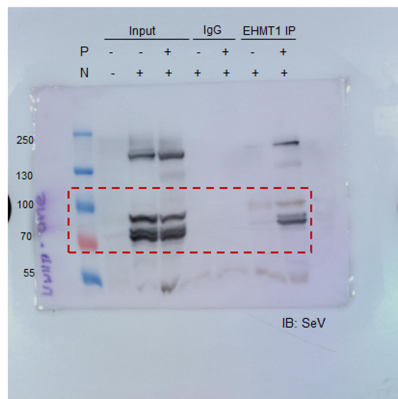

**6G**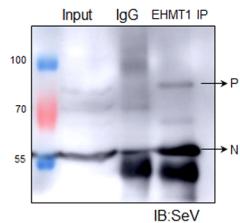**6H**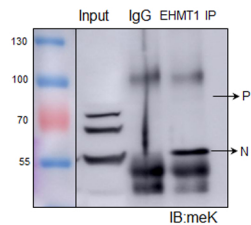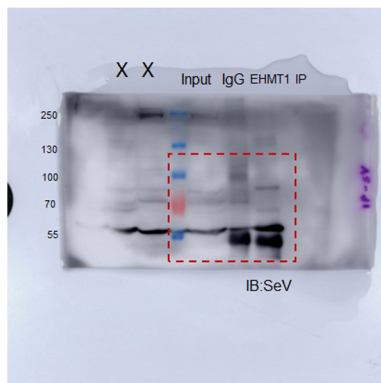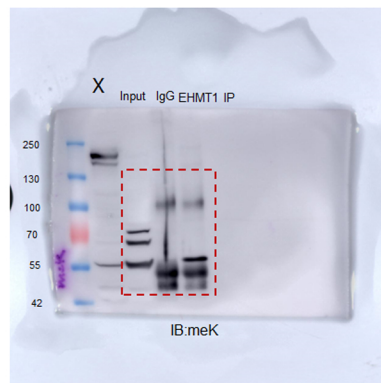

61

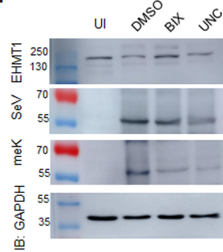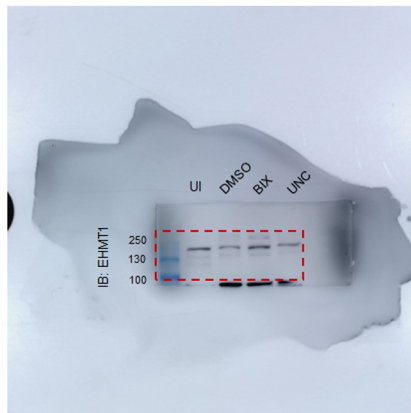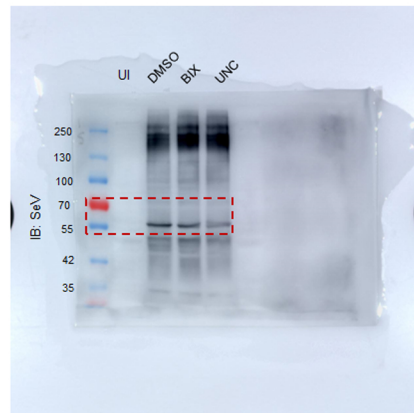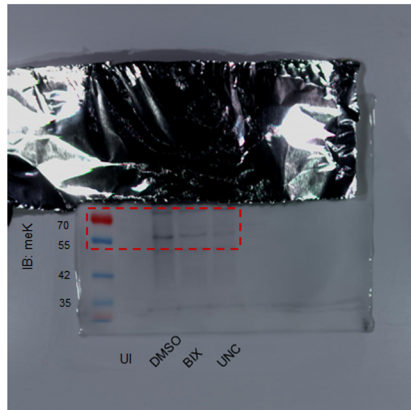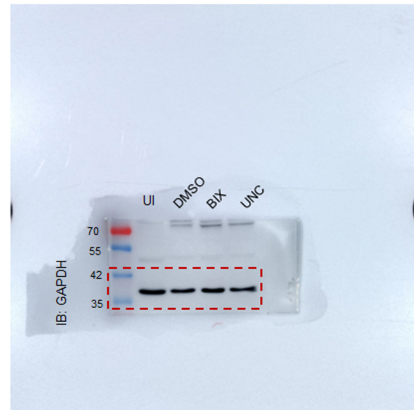

7J

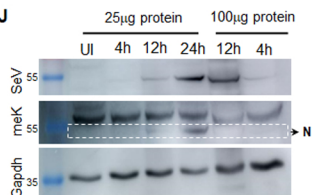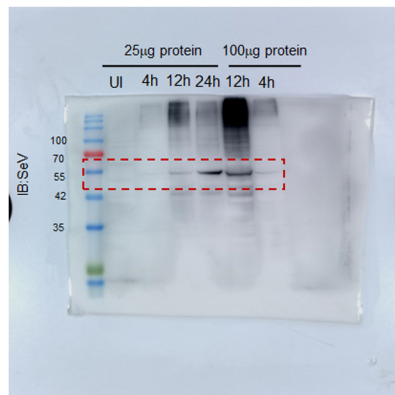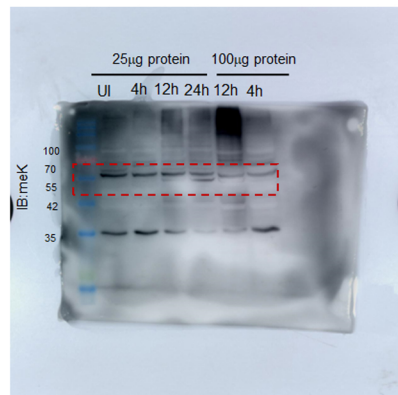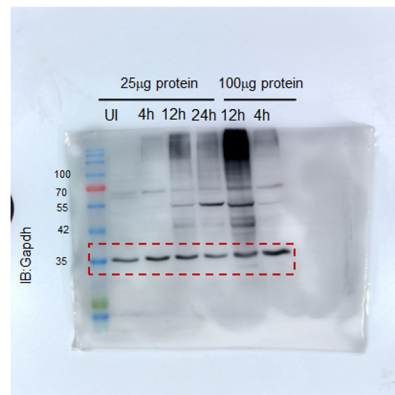

**S3A**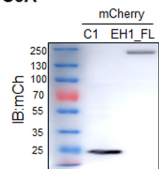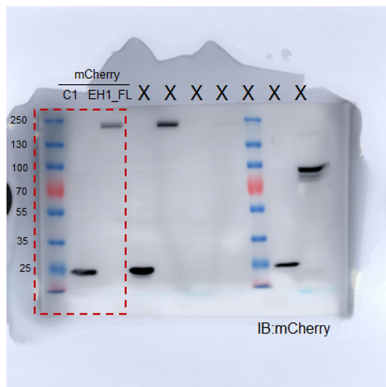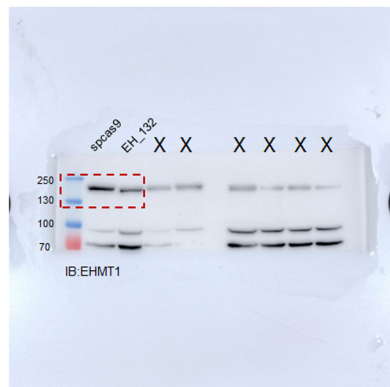**S5C**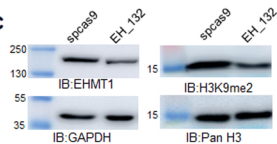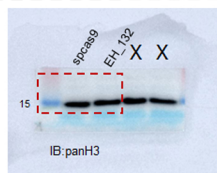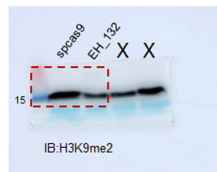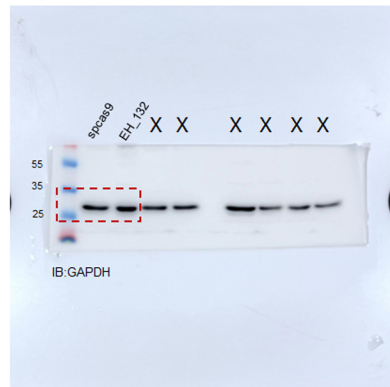

S5D

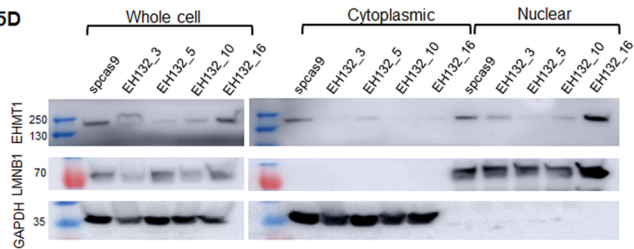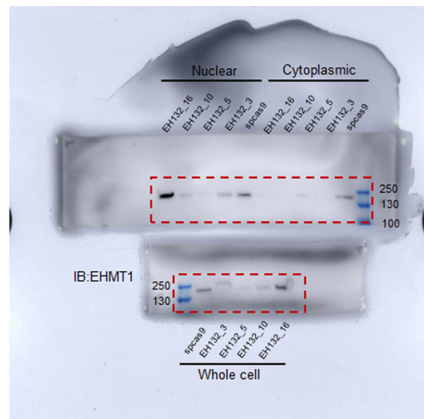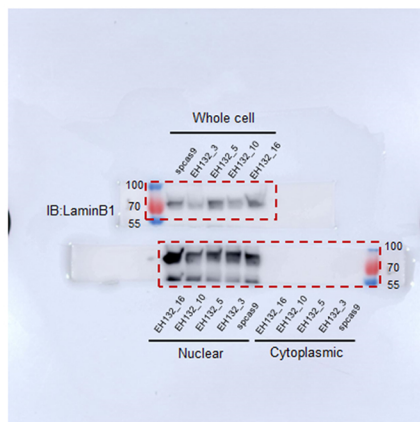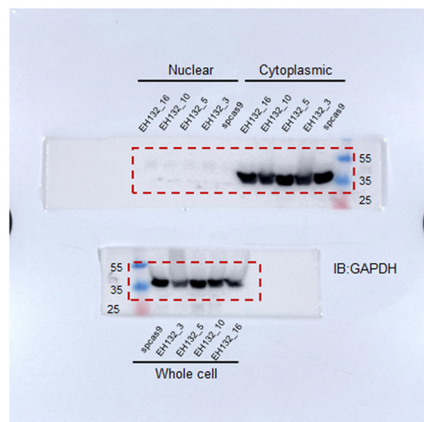

## S6A

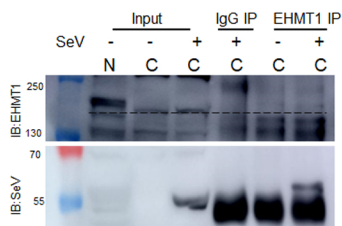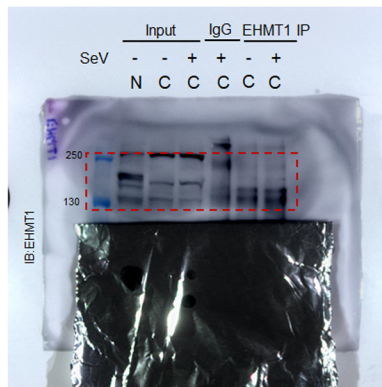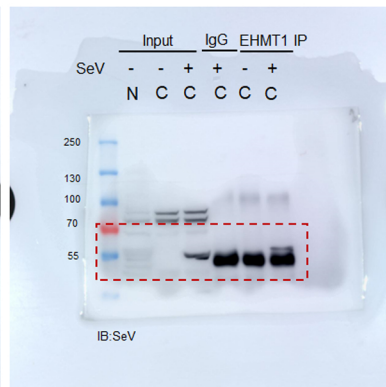

## S6B

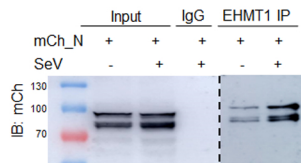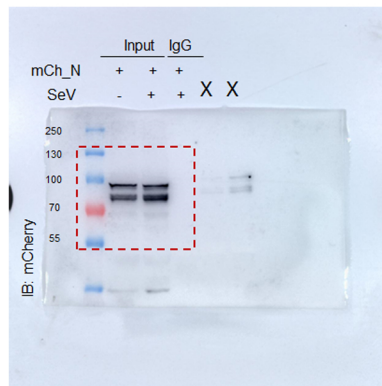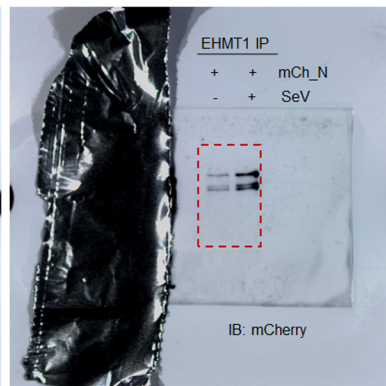

## S6C

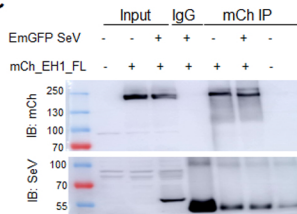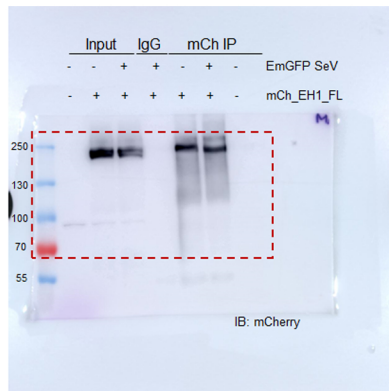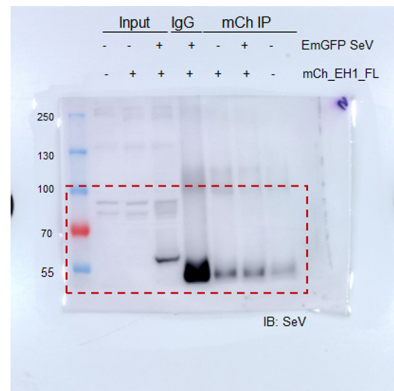

## S6D

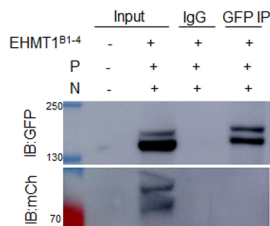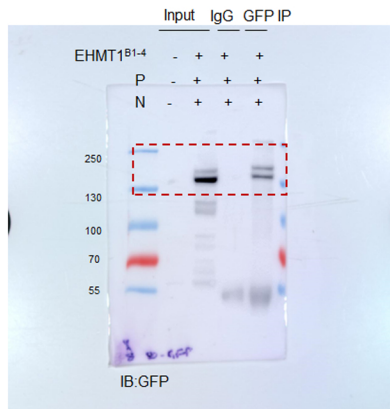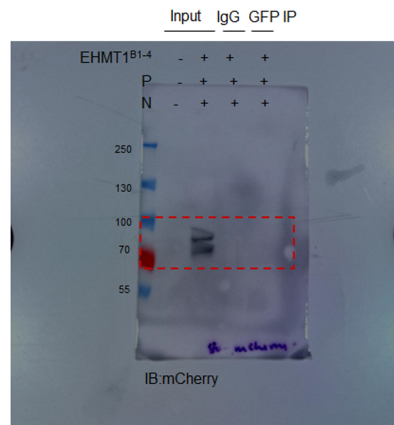

**S6E**

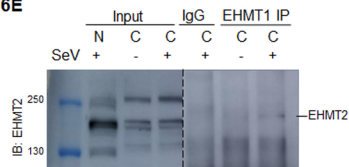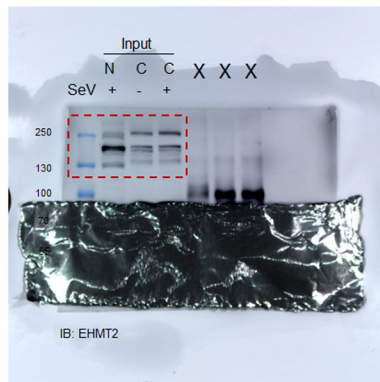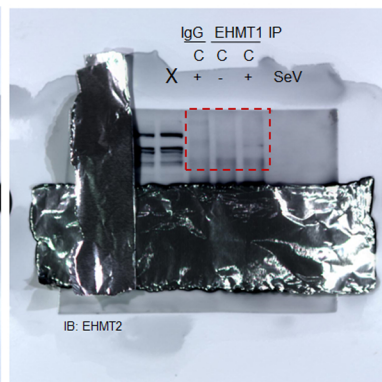

**S6F**

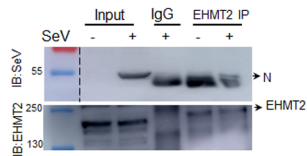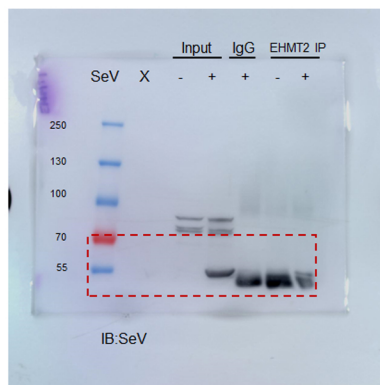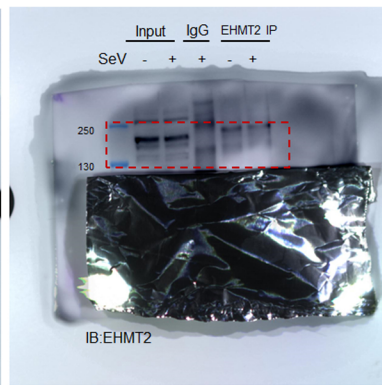

**S6I**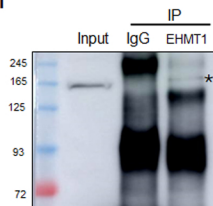

IB: EHMT1

**S6J**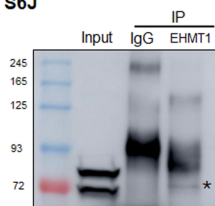

IB: mCh

**S6K**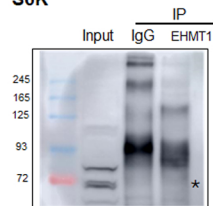

IB: meK

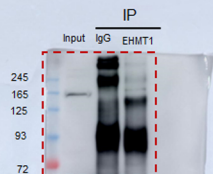

IB: EHMT1

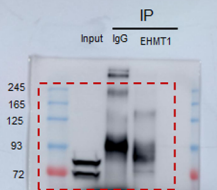

IB: mCherry

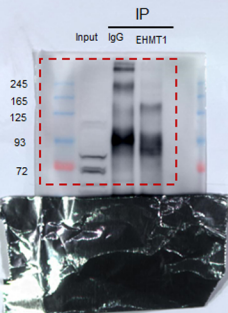

IB: meK

Supplement: S1 Raw Images — (PDF) [file pbio.3002871.s010.pdf]
