# Supplementary material for: A cytoplasmic form of EHMT1N methylates viral proteins to enable inclusion body maturation and efficient viral replication
Source: PLoS Biol. 2024 Nov 7;22(11):e3002871. doi: 10.1371/journal.pbio.3002871 (PMC11575796; doi:10.1371/journal.pbio.3002871)
Supplement: Attachment — Submitted filename: Rebuttal 230724.pdf [file pbio.3002871.s012.pdf]

# Novel nucleo-cytoplamic form of Euchromatic Histone Methyltransferase1 (EHMT1$^{N/C}$) methylates RNA viral proteins involved in inclusion body formation to facilitate viral replication

Kriti Kestur Biligiri [1, 2], Nishi Raj Sharma[3], Abhishek Mohanty[4], Debi Prasad Sarkar[5], Praveen Kumar Vemula[4], Shravanti Rampalli [1,2 *]

Reviewer#1: In this manuscript, Biligiri et al. reported that a novel nucleo-cytoplamic form of EHMT1, which is a histone methyltransferase, can interact with N and P protein of the Sendai virus and promote inclusion body formation upon virus infection. They explored the underlying mechanism and suggested a new mechanism by which cytoplasmic EHMT1 acts as proviral host factor that regulates host-pathogen interface to discern RNA viral pathogenesis through phase separation (or condensation) of the inclusion bodies. This new discovery is interesting and provided the community to appreciate the association between histone methyltransferase and virus inclusion bodies. There are some concerns needed to be addressed so their conclusion can be more solid:

***Major concern 1:*** *The authors could try 1-2 more virus to infect the cells and see whether EHMT1 still can form condensates.*

**Response:** We thank the reviewer for this suggestion. Inclusion body formation is a shared feature among RNA viruses ([1], [2], [3], [4]). Therefore, we tested if the condensation of EHMT1 is conserved in response to infections caused by pathogenic single stranded RNA viruses [Chandiura virus (CHPV) and Dengue Virus (DenV)] that are human pathogens. Below are the details of our investigations:

1.<u>Chandipura Virus (CHPV):</u> CHPV is a negative-strand RNA virus and an emerging tropical pathogen which forms cytoplasmic inclusion bodies (IBs) [5]. Infection of MEFs with CHPV and immunolabelling with EHMT1 and CHPV L protein was performed at 5h p.i.. CHPV condensates labelled with CHPV_L antibody indicated that CHPV IBs did not resemble SeV IBs but had a morphology of spherical speckle-like structures and were composed of heterogenous sizes. Interestingly, EHMT1 formed condensates in the cytoplasm (Fig. R1A, Fig. 9A), where it was found to colocalize specifically with large ChPV IBs labelled with CHPV_L (Fig. R1A, Fig 9A).

2.<u>Dengue Virus (DenV):</u> We also employed DenV, a single strand positive sense RNA virus, a member of Flaviviridae family, to examine if EHMT1 condensates in response to DenV Infection. The DenV Non-Structural protein 1 (NSP1) is a part of the viral replication complex, also a marker of DENV replication organelles, known as Vesicle packets [6], [7]. HepG2 cells infected with Dengue virus were immunolabelled with EHMT1 and Nsp1 parallelly at 24h and 48h post infection (Fig R1B and C, Fig. 9B and C). DenV infected cells marked with Nsp1 demonstrated a reticulate pattern of staining (invaginations from the Endoplasmic reticulum) along with a few punctate-like structures (Fig R1B, Fig. 9B). Infected cells labelled with EHMT1 also demonstrated a similar punctate pattern of staining in the cytoplasm, which partly resembled the pattern of Nsp1 staining (Fig R1C, Fig. 9C). EHMT1 as well as NSP1 antibodies were raised in rabbit, therefore we were unable to perform co-immunolabelling.

Altogether, these data indicated that the recruitment of EHMT1 into viral IBs is not unique to Sendai virus infection but may be a broad response to multiple types of viral infections. This data is included in Figure 9 and Result ‘Cytoplasmic condensation of EHMT1 is conserved

among other members of the single strand RNA virus family' of the revised manuscript.

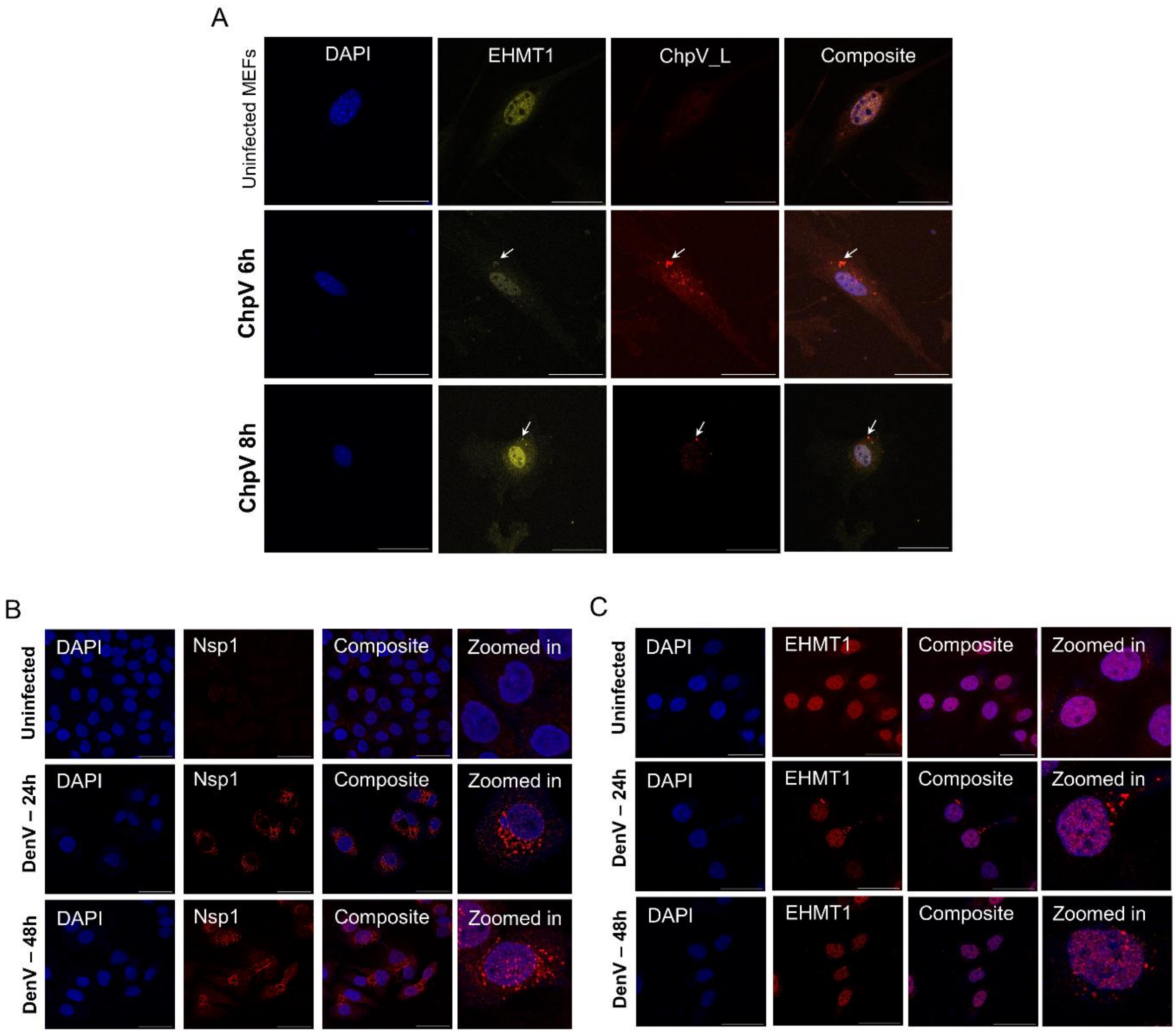


Reference Figure 1: Confocal microscopic images of (A) MEF infected with ChPV at 6h and 8h p.i., co-immunolabelled with EHMT1 (yellow) and ChPV_L (red). Composite images are with DAPI (blue) stained nuclei. (B) HepG2 cells infected with DenV immunolabelled with Nsp1 (red) and (C) EHMT1 (red), composite images are with DAPI (blue) stained nuclei.

***Major concern 2:*** *Does EHMT2 also can form condensates upon virus infection?*

**Response:** EHMT1 and EHMT2 are known to form functional heterodimers; since EHMT2 has a cytoplasmic isoform, we tested the recruitment of EHMT2 to SeV IBs by Immunofluorescence experiments. Immunolabeling for EHMT2 in SeV infected cells did not indicate the presence of EHMT2 in IBs (Fig R2A, fig. S1G). We speculated that this could either be due to the absence of EHMT2 in SeV IBs or EHMT2 could be present in a non-

natively folded state, leading to burial of its antibody-specific epitope, due to which the antibody probably didn't recognize or bind to EHMT2 in the IBs.

In order to negate any possible technical concerns, first we performed immunoprecipitation of EHMT1 from the cytoplasmic fraction of SeV infected cells and found an association with EHMT2 (fig. S6E), indicating that the two proteins associate with each other in the cytoplasm of cells infected with SeV. Assuming that cytoplasmic EHMT1 is mostly concentrated in viral IBs, there is a high chance that association of EHMT1 with EHMT2 in the cytoplasm of infected cells could be stemming from viral IBs. To determine whether EHMT2 associates with SeV IBs in the cytoplasm of infected cells, we immunoprecipitated EHMT2 from the cytoplasmic fraction of infected cells and found an association with SeV N protein (Fig R2B, Fig. S6F). These experiments confirmed the association of EHMT2 with the viral Nucleoprotein. This data is included in Supplementary figure 1H, Supplementary figure 6E and 6F of the revised manuscript.

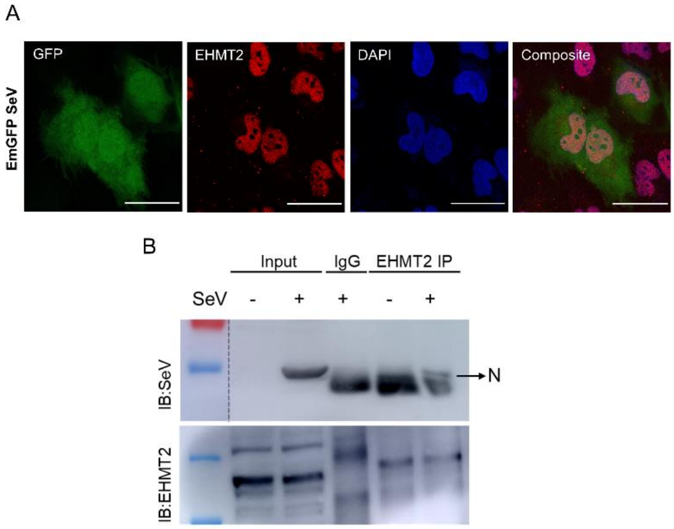


Reference Figure 2: (A) Confocal microscopic images of BEAS-2B cells infected with EmGFFP reporter SeV (green), immunolabelled with EHMT2 (red); nuclei are stained blue with DAPI. (B) Western blotting of EHMT2 immunoprecipitated elute, probed with antibodies against SeV (top) and EHMT1 (bottom).

***Major concern 3:*** *Does EHMT1 or the N, P proteins has IDR domain (intrinsically disordered regions)? And What is their function?*

**Response:** Protein database indicates that 55% of N-terminal region of EHMT1 comprises of disordered regions. The N-terminal half of EHMT1 doesn't have any functional domains but

is believed to provide structural stability to EHMT. Activity of EHMT1 activity lies in the Ank and SET domain, which reside in the C-terminal half of the protein [8], [9].

Similarly, 62% of the Sendai viral Phosphoprotein comprises of disordered regions. The Phosphoprotein is a cofactor of the viral RNA-dependent RNA polymerase that aids in viral replication and transcription [1], [2], [10]. It also chaperones nascent Nucleoprotein, which encapsidates the viral RNA [11]. The SeV Nucleoprotein, however, has a very minute proportion of disordered region. When the Nucleo- and Phosphoprotein interacts with each other, they have the intrinsic property of forming liquid phase separated condensates in-vitro or in the cytosol of the host [12]. As their concentration increases, they enlarge in size and recruit necessary host proteins. We demonstrated this phenomenon for SeV by co-transfecting cells with the N and P proteins, where EHMT1 was also found to get recruited (Fig. 2E). Any protein further recruited (like the host protein, EHMT1) to these liquid condensates, also exhibit condensate-like behaviour because they belong to the same complex.

***Major concern 4:*** *Figure 3, The authors used IP-EHMT1 to pull down the N and P proteins; could they use IP-N or IP-P to pull down the EHMT1 protein?*

**Response:** In our IP EHMT1 experiments performed on SeV infected cells, we were able to detect an interaction with N protein predominantly and a weak interaction with P protein. Since N+P co-transfection was both responsible and sufficient to recruit EHMT1 to IBs (Fig. 2E), we examined the nature of interaction of EHMT1$^{N/C}$ independent of infection. Here, we transfected cells either with N or both N+P; IP EHMT1 from the cytoplasmic fraction indicated a strong association with N only in the co-transfected system (Fig. R3A, Fig. 6E). This experiment revealed that EHMT1$^{N/C}$ associated with N protein in a system where IBs are formed either by infection or co-transfection (Fig. 6E and G)To address the reviewer's concern in a direct manner we studied interaction between N proteins with the nucleo-cytoplamic forms of EHMT1 identified (EHMT1$^{B1-3}$_EGFP or EHMT1$^{V09}$_EGFP). We triple transfected the cells with mCh_N + piRFP_P and either EHMT1$^{B1-3}$_EGFP or EHMT1$^{V09}$_EGFP. These two isoforms of EHMT1 were found to be the specific Nucleo-cytoplasmic forms that exhibit distinct localization pattern, get recruited to IBs and associate with the Nucleoprotein (Fig. R3B and C, Fig. 3C, E, F and G, 6A and B). mCherry IP to immunoprecipitate N was performed on the cytoplasmic fraction of triple transfected cells, the elute was western blotted and probed with GFP to determine any association with the EHMT1's nucleo-cytoplasmic isoforms (Fig.

R3D and E, Fig, 6C and D). The blot probed with GFP indicated an association of the Nucleoprotein with both EHMT1$^{B1-3}$ as well as EHMT1$^{V09}$. Thus, we were able to demonstrate the association between nucleocytoplasmic forms of EHMT1 and N by Immunoprecipitation.

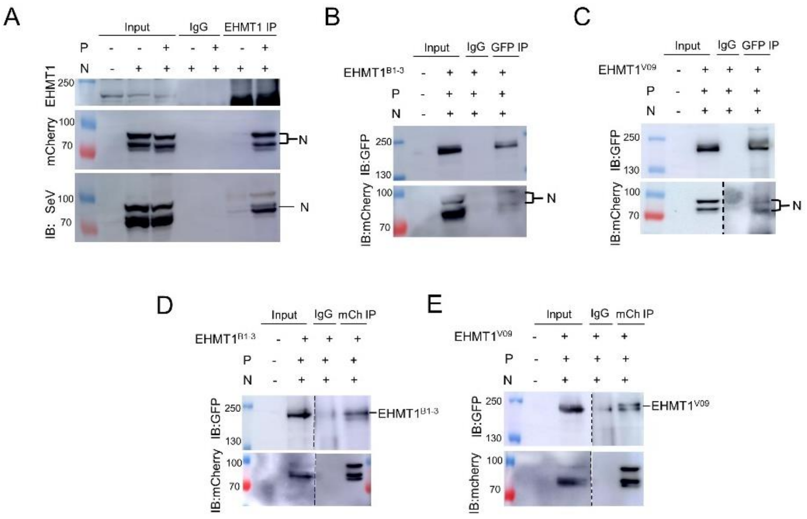


Reference figure 3: Western blotting of Input and Immunoprecipitated elute from (A) EHMT1 IP performed on the cytoplasmic fraction of cells transfected either with mCh_N or N+P, blots probed with EHMT1, mCherry and SeV. (B) and (C) GFP IP performed on the cytoplasmic fraction of cells co-transfected with N+P along with either (B) EHMT1$^{B1-3}$_EGFP or (C) EHMT1$^{V09}$_EGFP, blots probed with GFP and mCherry. (D) and (E) mCherry IP performed on the cytoplasmic fraction of cells co-transfected with N+P along with either (B) EHMT1$^{B1-3}$_EGFP or (C) EHMT1$^{V09}$_EGFP, blots probed with GFP and mCherry.

***Major concern 5:*** *Please mark the protein size (i.e. kilodalton) in the Western blots.*

**Response:** We thank the reviewer for this suggestion. We have marked the Western blots in the revised manuscript.

***Major concern 6:*** *Why the full-length vector of EHMT1 cannot enter the cytoplasmic? Can the authors give any explanations?*

**Response:** The full length EHMT1 is a nuclear protein whose expression is restricted to the nucleus (Fig. S3C). This form has predicted NLS sequences, which could be responsible for its localization and restriction to the nucleus.

In the current study, we have characterized isoforms of EHMT1 from public databases. Among them, EHMT1 isoforms Q9H9**B1-3 and** Q9H9**B1-4** were missing 1185-1298 and 809-1298 regions respectively. We also noticed an isoform (A0A1B0G**V09**) that was alternatively spliced with initial 93 missing bases, where exons 1 and 2 were completely spliced out and exon 3 was partly spliced out. The $94^{th}$ base had an in-frame ATG, which was the start site of this isoform; the rest of the transcript aligned 100% with the full-length form. To identify the subcellular localization of EHMT1 isoforms we cloned the above isoforms in an EGFP_N1 reporter expression vector. We termed these constructs $EHMT1^{B1-3}$_EGFP; $EHMT1^{B1-4}$_EGFP and $EHMT1^{V09}$_EGFP. The above-mentioned constructs were transfected into HEK and visualized for the GFP expression in nuclear vs. cytoplasmic compartments. All the isoforms cloned demonstrated predominant nuclear localization signal similar to canonical full length protein (Fig. 3C to E). Interestingly about 45%-50% of cells transfected with $EHMT1^{B1-3}$_EGFP or $EHMT1^{V09}$_EGFP demonstrated a faint signal in the cytoplasm (Fig. 3C and E). However, such cytoplasmic signal was absent in cells transfected with $EHMT1^{B1-4}$_EGFP construct (Fig. 3D). Taken together, all the isoforms studied had preferential localization to the nucleus and promiscuous cytoplasmic localization when a portion of EHMT1 from either the N-terminal ($EHMT1^{V09}$) or C-terminal ($EHMT1^{B1-3}$) was missing.

While studying the domains of EHMT1 that are responsible for retention of EHMT1 in the nucleus, we also identified that the loss of 500 amino acids (from exon 5-13) also showed promiscuity of EHMT1 to move to cytoplasm (fig. S3 I and J). Our data from EHMT1 isoforms and domain deletions (delta EHMT1), where small regions of EHMT1 were missing either from the ends of C-terminus, N-terminus or the middle demonstrated altered distribution of EHMT1. From the above studies, we could not pinpoint onto a particular region whose loss renders cytoplasmic localization of EHMT1. Thus, we currently don't understand the mechanism by which full-length EHMT1 is retained in the nucleus or the regions that impart distinct subcellular localization to the nucelo-cytoplasmic forms of EHMT1. From our data, we speculate that the localization properties of EHMT1 is determined mostly by its protein folding state, which requires structural biological studies to conclusively understand its nature. Such studies are currently beyond the scope of this manuscript.

***Major concern 7:*** *The reviewer is wondering that Delta EHMT1 is indeed existed in the real-world? Can the authors give and evidences? What is ratio of Delta EHMT1 vs full-length EHMT1? Who is responsible for the making of Delta EHMT1*

**Response:** Delta-EHMT1 doesn't exist in the real-world, this construct was cloned in the lab with the intent to understand the behaviour of various domains of EHMT1 protein in terms of their subcellular localization and association with viral IBs. Delta EHMT1 was missing regions between exons 5 - 13 of EHMT1, and upon transfection, demonstrated nucleo-cytoplamic localization. Since delta-EHMT1 is not encoded by cells and expressed only upon transfection, we cannot comment about its ratio in comparison with the full-length EHMT1, which is an endogenous protein.

However, as mentioned in response 6 and Fig. 3C to G, Fig. 6A to D of the manuscript, we have now identified the nucleo-cytoplasmic isoforms of EHMT1, which were previously deposited on databases. These forms, however, were uncharacterised; we cloned these forms and found that 2 of the forms Q9H9**B1-3** and A0A1B0G**V09** exhibit nucleo-cytoplasmic localization pattern, get recruited to IBs and associate with the viral Nucleoprotein.

***Major concern 8:*** *Did the two inhibitors specifically targeting EHMT1, can the authors give more evidences?*

**Response:** EHMT1 and EHMT2 share 80% sequence identity with their SET domain, the domain responsible for catalysing methylation of lysine residues on substrate proteins. BIX01294 and UNC0642 are two widely used inhibitors with specificity towards both EHMT1 and EHMT2.

BIX01294 binds the substrate binding region of EHMT1, thus preventing association of the substrate with the SET domain of the enzyme, EHMT1. BIX01294 was found to inhibit G9a/EHMT2 with an $IC_{50}$ value of 1.7μM and 38μM for GLP/EHMT1 [13]. When examined for their specificity, BIX01294 did not inhibit any other histone methyltransferases even at a concentration of 45mM [14]. UNC0642 is a competitive inhibitor against EHMT1 and EHMT2, with an $IC_{50}$ of <15nM and selectivity of >100 fold as tested with over 13 other Histone methyltransferases [15].

Nevertheless, data obtained by depleting EHMT's enzymatic activity using the inhibitors or guide RNA mediated specific depletion of EHMT1 levels both yielded similar results, indicating that EHMT1 was responsible for the processes governed during viral infection.

***Major concern 9:*** *In respect to RNA virus replication, the authors only used the gRNA as the readout. They should give more readouts since virus replication is the most important and the only function assay in this study.*

**Response:** We thank the reviewers for raising this point, we have now performed Haemolysis assay as a functional assay to validate our claim. The Sendai viral Hemagglutinin Neuraminidase (HN) protein is known to bind sialic acid receptors on the cell surface, facilitating viral attachment and entry into the host. When bound to RBCs, HN proteins can disrupt the cell membrane, inducing their lysis, which results in release of haemoglobin. Release of haemoglobin is proportional to the viral load, thus enabling us to quantify viral replication across various conditions. We thus employed this assay to assess viral replication upon inhibition of EHMT1's enzymatic activity [16].

BEAS-2B cells were infected with WT SeV and treated with EHMT inhibitors, BIX and UNC. The supernatant was collected 24h p.i. and incubated with 1% RBCs on ice for attachment, followed by incubation at 37°C for lysis of RBCs. The cells were then centrifuged and supernatant containing haemoglobin from lysed RBCs were quantified; the amount of lysis being directly proportional to the viral load. From this, we observed that haemolysis was reduced by about 50-60% in EHMT inhibitor treated cells compared to the untreated infected cells (Fig R4, Fig. 4E and F). This indicated and further strengthened our claim that EHMT1's enzymatic activity is essential for viral replication. This data had been included in the revised manuscript in lines 336-347.

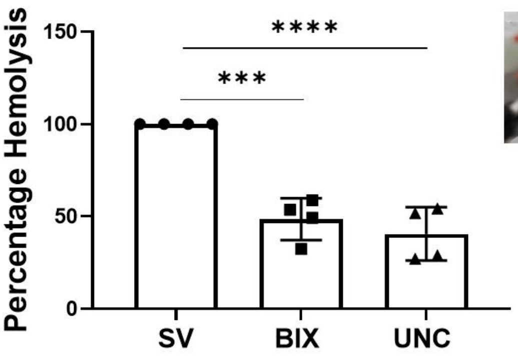


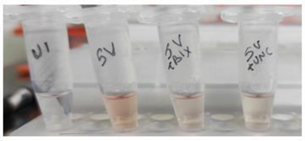

Reference figure 4: Graph representing the percentage of hemolysis upon SeV infection +/- EHMT inhibitors. Image representing the supernatant prior to measurement of absorbance.

***Major concern 10:*** *For Figure 5 and Figure 7, the authors could provide an excel file to include their original date obtained from their microscope observation on inclusion bodies numbers and sizes.*

**Response:** Excel sheet is attached along with this document.

***Major concern 11:*** *For Figure 6, the authors should perform in vitro experiment to confirm that EHMT1 can demethylate N protein.*

**Response:** Invitro experiment requires us to purify recombinant Nucleoprotein and EHMT1's SET domain and incubate the two proteins with necessary components to determine whether EHMT1 is capable of methylating N. However, several issues have precluded us from performing this experiment:

Methylation of Nucleoprotein was demonstrated exclusively in SeV infected cells. In cells co-transfected with N and P alone, IBs were formed and EHMT1 was recruited to these structures. However, EHMT1 did not methylate N protein in absence of viral infection. This experiment indicated that methylation of the Nucleoprotein requires more than just the recruitment/association of EHMT1. We believe that EHMT1 mediated N protein methylation probably needs other factors which could be viral, host derived or a combination of both, which are present in the IBs upon infection. Thus, performing an in vitro methylation experiment with just with the Nucleoprotein and EHMT1's SET domain may not be sufficient to address the reviewer's concern.

***Major concern 12:*** *Can the authors provide any in vivo data to support their discovery?*

**Response:** Sendai virus is a murine parainfluenza virus, known to be transmitted via respiratory droplets. Several decades ago, Sendai viral infection had caused mortality in rodents like mice, rats and hamsters [17]. Thus, to perform in vivo experiments, we require a specialized and dedicated BSL3 facility, isolated from the rest of the animal house. Since we aren't equipped with such a facility, it's not feasible for us to perform in vivo experiments for this manuscript. Limitations such as these have prevented investigators from using this or other viruses in animal models.

***Major concern 13:*** *All of the virus infection experiment is only last 1-3 days, the reviewer is wondering can we see the same phenomena in a stable infection situation?*

**Response:** A survey of the literature reveals that research articles from the field of viral Inclusion Bodies have either used Wild Type or recombinant virus infected cells to study the regulation of IBs during early to peak stages of infection. In alignment with this, we employed a similar model to study IBs formed by Sendai Virus. While we do not understand how stable infection will help us improve our findings, the EmGFP Sendai virus/OSKM Sendai virus used for this study persists in cells for about 10 days, which can be detected by the presence of Inclusion bodies, marked by EHMT1. However, since the virus attains its peak around 1-3 days, we performed all the experiments around this time window, but we have results to state that our findings of EHMT1 being recruited to IBs hold true for about 10 days (Fig R5A and B). We are providing this data below with the hope that it will address the reviewer's concern.

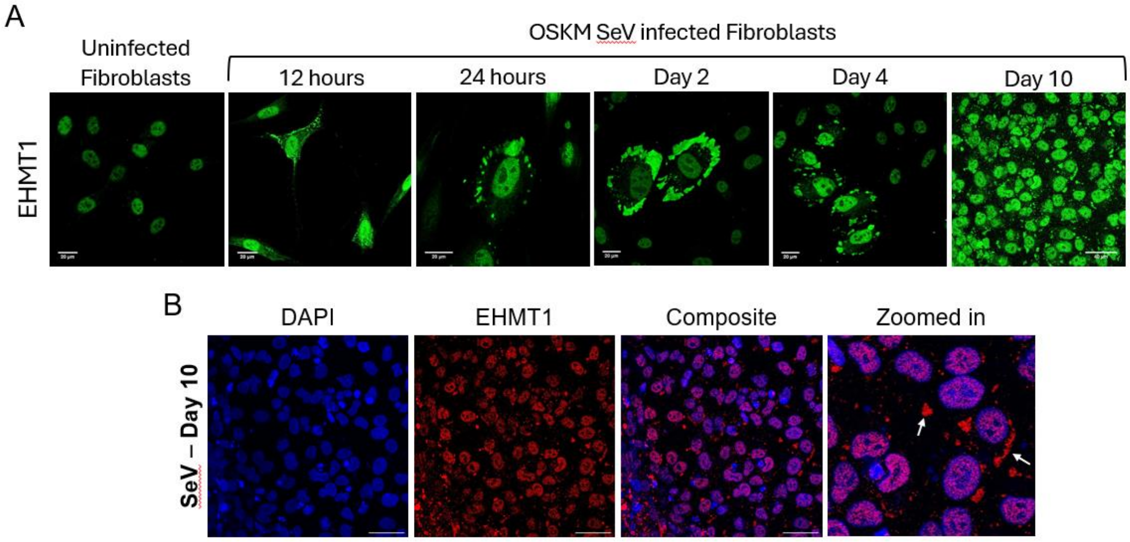


Reference figure 5: Confocal microscopic images of Fibroblasts uninfected/infected with Sendai virus (A) immunolabelled with EHMT1 (green) from 12h to Day10 post infection. (B) immunolabelled with EHMT1 (red), nuclei stained with DAPI (blue) to distinguish between the nuclear and cytoplasmic EHMT1.

**Reviewer #2:**

**In this article, the authors describe the recruitment of an EHMT1 methyltransferase to Sendai virus IBs. This recruitment increases as the virus cycle progresses and coincides with an increase in the size of IBs. The authors show that EHMT1 is a proviral factor. EHMT1 interacts with the RNPs of the virus. It would methylate the N and P of Sendai virus. Unfortunately, the experiments need to be completed to support the conclusions presented by the authors. In addition, the manuscript needs extensive restructuring to make it more readable. Firstly, a lack of rigour in the experiments and/or in the presentation of the results limits the interpretation of the results and the soundness of the conclusions. In particular, data presented are not strong enough to support results 3, 4 and 6.**

***Major concern 1:*** *Result 1 (lines 104-181): EHMT1 is recruited to Sendai IBs and pseudo-IBs generated by expression of N and P. Infection of different cell types with Sendai virus (wild-type and EGFP) showed translocation of EHMT1 to IBs. The same was observed in pseudo-IBs formed with N and P alone. The first 2 paragraphs are redundant, and the figures are not very informative. The uninfected panel is missing from Figures 1 and 2 for full interpretation. The figures should be combined into a complete and coherent main figure and one or two sup-figures.*

**Response:** EHMT1 was first identified about 3 decades ago in a transcriptional repressor complex, where it was found to methylate histones and serve as a transcriptional regulator. Since then, most of the articles have described its role as an epigenetic regulator in various biological contexts; where it was regarded and/or reported as a protein restricted to the nucleus. In this study, we had a serendipitous discovery where we happened to identify this protein in the cytoplasm. Since this was the first study that identified EHMT1 in the cytoplasm, we explained in Figure 1 about the way we happened to identify this protein in the cytoplasm. Figure 1 also described additional experiments performed by us to conclusively demonstrate how cytoplasmic condensation of EHMT1 was not in response to reprogramming but induced by the vector employed for the process of reprogramming. Considering the field of epigenetics, where this protein is strongly believed to be a nuclear protein, we think that it is necessary to explain how we happened to discover this protein in the cytoplasm for the first time.

Sendai viral IBs were not characterised until now and since this study focuses on SeV IBs, we performed characterization experiments with appropriate markers to demonstrate that EHMT1

gets recruited to SeV IBs upon infection. In figure 2, we have also identified the necessary factors responsible for the recruitment of EHMT1 to cytoplasmic IBs.

While the first figure describes about our serendipitous discovery and our path towards finding the causative factor, the second figure characterizes the condensates identified in the cytoplasm. Thus, we believe that keeping the figures independent would help delivering a clear message to the readers. However, if the reviewer still insists, we would be willing to combine the two figures.

The uninfected panel for Figures 1 and 2 is provided below (Fig R6) and included in the revised manuscript (fig. S1A, D and E).

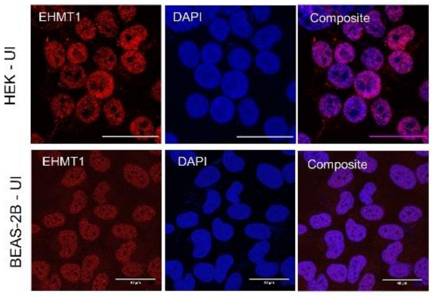


Reference figure 6: Confocal microscopic images of uninfected (UI) HEK (top) and BEAS-2B (bottom), immunolabelled with EHMT1 (red), nuclei counterstained with DAPI (blue).

***Major concern 2.0:*** *Result 2 (lines 182-251): A distinct form of EHMT1 associates with IBs. The authors show a band of lower MW than EHMT1 recognised by the antibody against EHMT1. Observation of the disappearance of this band under conditions of EHMT1 under-expression would complete the demonstration. Note that this decrease is observed in Sup Fig 4D, but on a less resolving gel.*

**Response:** As the reviewer mentioned, the two distinct bands can be clearly visualised on a 4% gel. But here, we demonstrate the depletion of EHMT1 in both nuclear and cytoplasmic fractions with the appropriate controls (LaminB1 and GAPDH respectively), which isn't possible on a 4% gel due to its limited resolution. Also, 4% gel is extremely flimsy making it difficult to quantify the levels of EHMT1 between control and knock out conditions. We hope that this clarification addresses the reviewer's concern.

However, to strengthen this claim, we have now characterized the nucleo-cytoplamic forms of EHMT1. Based on our data from Western blotting of the Nuclear and cytoplasmic fractions,

we speculated that such small size difference as seen between EHMT1$^{N}$ and EHMT1$^{N/C}$ are usually a resultant of PTMs like phosphorylation or due to the formation of an alternative transcript by deletion of small exons from the full-length mRNA. Upon analysis of EHMT1 transcripts deposited on Uniport, we noticed three isoforms of the canonical full-length form of EHMT1 (Q9H9**B1**-1298 AA long). Among them, Q9H9**B1-2** codes for the first 67 amino acids of the canonical protein; the product thus formed is unstable. Q9H9**B1-3** and Q9H9**B1-4** were missing 1185-1298aa and 809-1298aa regions respectively. We also noticed an isoform (A0A1B0G**V09**) that was alternatively spliced with initial 93 missing bases, as a resultant of splicing events which resulted in the elimination of the first two exons and a part of the third exon. The 94$^{th}$ base had an in-frame ATG, which was the start site of this isoform; the rest of the transcript aligned 100% with the full-length form. To uncover the form of EHMT1 that exhibits cytoplasmic localization and associates with SeV Inclusion Bodies, we cloned all the above isoforms (except Q9H9**B1-2**) in EGFP_N1 reporter expression vector. We termed these constructs EHMT1$^{B1-3}$_EGFP; EHMT1$^{B1-4}$_EGFP and EHMT1$^{V09}$_EGFP. The above-mentioned constructs were transfected in HEK and visualized for GFP expression in nuclear vs. cytoplasmic compartments (Fig. R7A to C, Fig. 3C to E). All the isoforms cloned demonstrated predominant nuclear localization signal like the canonical full-length protein (Fig. R7A to C, Fig. 3C to E). Interestingly, about 45%-50% of cells transfected with EHMT1$^{B1-3}$_EGFP or EHMT1$^{V09}$_EGFP displayed a faint signal in the cytoplasm as well (Fig. R7B and C Fig. 3C, E, fig. S3E). However, such cytoplasmic signal was absent in cells transfected with EHMT1$^{B1-4}$_EGFP construct (Fig. R7A, Fig. 3D, fig. S3E). Upon transfection with mCh_N + piRFP_P, EHMT1$^{B1-3}$_EGFP as well as EHMT1$^{V09}$_EGFP were found to get recruited to SeV IBs (Fig. R7E and F, Fig. 3F and G). Taken together, these isoforms seemed to have a preferential localization to the nucleus along with cytoplasmic localization, similar to endogenous EHMT1. CRISPR/Cas9 mediated gRNA designed to deplete EHMT1 targets all the newly identified isoforms. This data is present in Fig. 5A and D of the revised manuscript.

***Major concern 2.1:*** *To show that this particular form of EHMT1 is recruited to IBs, the authors rely on 2 results: 1) Immunocapture of N and P by anti-EHMT1 in the cytosolic extracts, where only this form should be present. For a more robust demonstration, parallel migration of nuclear fractions (on a 4% gel) is required.*

**Response:** Immunoprecipitation requires us to elute protein from antibody-conjugated Dyna beads (Magnetic beads used for IP). During elution, the antibody bound to the beads also gets eluted into the mix, which is then resolved on an SDS-PAGE gel. 4% gel doesn't have the ability to resolve proteins especially in IP samples, where EHMT1 (170KDa) and IgG (55KDa) bands need to be resolved to interpret the results. When we resolved these proteins on a 4% gel, the IgG and EHMT1 bands were at the same level as shown below, which defeated the purpose of using a 4% gel. Hence, we resolved fractions on an 8% gel and further resolved them until all the bands below 55KDa had run out of the gel. On doing so, we were able to resolve the nuclear EHMT1 into two distinct bands (Fig. R7G, Fig. S6A). Here, the cytoplasmic band in the input correlated with the cytoplasmic EHMT1 IP'ed band, thus demonstrating that EHMT1$^{N/C}$ is indeed the protein associating with the SeV Nucleoprotein (Fig. R7G, Fig. S6A).

To strengthen this claim, we identified the nucleo-cytoplasmic isoforms of EHMT1, which gets recruited to IBs (as described in response 2.0), we performed IP experiments with the EHMT1 isoforms. The full-length EHMT1, tagged with mCherry, mCh_EHMT1_FL did not associate with the viral proteins as mentioned previously in the manuscript (Fig. R7H, Fig. S6C). EHMT1$^{B1-4}$ also did not exhibit nucleo-cytoplasmic localization or associate with SeV N (Fig. R7A, D and I, Fig. 3D and H, fig. S6D). However, we co-transfected cells with mch_N and piRFP_P along with either EHMT1$^{B1-3}$_EGFP or EHMT1$^{V09}$_EGFP and isolated the cytoplasmic fraction. Immunoprecipitation using GFP specific antibody followed by western blotting demonstrated an association of EHMT1 isoforms with the nucleoprotein (Fig. R7J and K, Fig. 6A and B). Reverse IP experiment further reiterated that the viral Nucleoprotein associated with both EHMT1$^{B1-3}$_EGFP as well as EHMT1$^{V09}$_EGFP (Fig. R7L and M, Fig. 6C and D)., which strengthened our claim that distinct nucleo-cytoplasmic forms of EHMT1 associate with the viral Nucleoprotein. This data is presented in the revised manuscript in Fig. 3C to H, Fig. 6A to D and Fig. S6A to D.

Overall, to address this concern, we have demonstrated parallel migration of nuclear and cytoplasmic fractions, where the IP'ed band corresponded with the cytoplasmic protein. Additionally, we demonstrated the specific forms of EHMT1 that associate with the Nucleoprotein.

***Major concern 2.2:*** *2) The authors used transient transfections with vectors expressing either the full protein or truncated forms. The localisation of these fluorescent proteins in uninfected*

*and infected cells was analysed by immunofluorescence. The results are presented in 3 different figures, making them difficult to read. The infection state is not tested for all mutants and only one cell is shown. There are no results showing that the smaller MW form is close to the delta EHMT1 form constructed by the authors. Although these results support the hypothesis put forward, they do not allow us to conclude on the nature of the N/C form of EHMT1.*

**Response:** In the previous version of the manuscript, we had employed a strategy where different domains of EHMT1 were cloned to determine the sequence responsible for nucleo-cytoplasmic expression pattern. However, we agree that this strategy did not allow us to conclude on the distinct form of EHMT1. Therefore, we employed an alternative strategy, as described in Response 2.0 and 2.1, where we identified and explained the isoforms EHMT1$^{B1-3}$, EHMT1$^{B1-4}$ and EHMT1$^{V09}$, cloned into EGFP. Among these, EHMT1$^{B1-3}$ and EHMT1$^{V09}$ exhibited Nucleo-cytoplasmic localization, co-localised into SeV IBs and associated with the Nucleoprotein (Fig. R7). From these experiments, we were able to conclude that two of the previously reported isoforms, EHMT1$^{B1-3}$ and EHMT1$^{V09}$ are now characterised in terms of their localization pattern; by doing so, we have been able to gain insights into the nucleo-cytoplasmic nature of EHMT1. This data is included in the revised manuscript in Fig. 3C to H, Fig. 6A to D and Fig. S6A to D.

While our data on specific expression of individual isoforms of EHMT1 helped us decipher the forms of EHMT1 that exhibit such distinct localization profiles, we found that Uniprot also reported twenty-four computationally predicted isoforms of EHMT1. A closer look at the deposited isoforms revealed that these transcripts encode for various lengths of EHMT1, encompassing either all or few domains. While it is not clear if these forms are indeed expressed in cells, we tested the localization profiles of various domains of EHMT1 into SeV IBs (Fig. S3I and J). We have also incorporated data for the Ank+SET expression in IBs in the revised manuscript (Fig. S3J). Our data from expression of various domains led us to identify their propensity to associate with SeV IBs.

We hope that our revised data demonstrating the nucleo-cytoplasmic forms of EHMT1 has addressed the reviewer's concerns. This data has been included in Results 3 and 6 of the manuscript under the sections 'A distinct nucleo-cytoplasmic form of EHMT1 associates with SeV IBs' and 'EHMT1$^{N/C}$ interacts with and methylates the SeV Nucleoprotein upon infection' respectively.

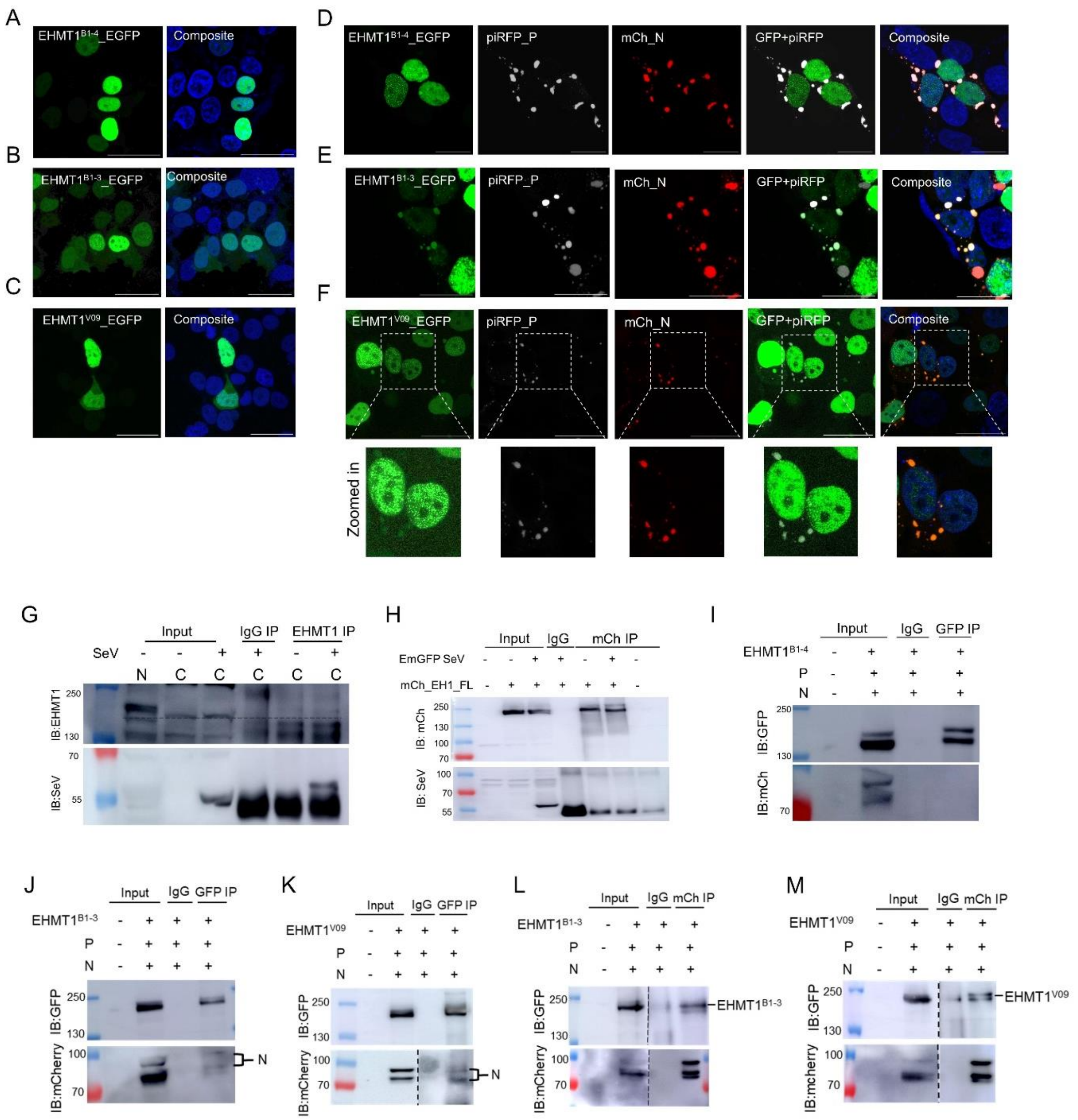


Reference figure 7: (A) to (C) Confocal microscopic images of HEK transfected with (A) EHMT1$^{B1-4}$_EGFP, (B) EHMT1$^{B1-3}$_EGFP and (C) EHMT1$^{V09}$_EGFP. (D) to (F) Confocal microscopic images of HEK transfected with mCh_N and piRFP_P along with either (D) EHMT1$^{B1-4}$_EGFP, (E) EHMT1$^{B1-3}$_EGFP or (F) EHMT1$^{V09}$_EGFP. Composite images are with DAPI (blue) stained nuclei. Scale bar - 40$\mu$m. (G) to (M) Western blotting of IP elutes and input from (G) EHMT1 IP performed on the cytoplasmic fraction of SeV uninfected or infected cells. Blots probed with EHMT1 and SeV (H) mCherry IP from the whole cell lysate of cells transfected with mCh_EHMT1_FL and infected with EmGFP SeV, blots probed with mCherry and SeV. (I) to (K) GFP IP from cytoplasmic fraction HEK triple transfected with mCh_N, piRFP_P and either (I) EHMT1$^{B1-4}$_EGFP, (J) EHMT1$^{B1-3}$_EGFP or (K)

EHMT1$^{V09}$_EGFP, blots probed with GFP and mCherry. (L) and (M) mCherry IP from cytoplasmic fraction of HEK triple transfected with mCh_N, piRFP_P and either (L) EHMT1$^{B1-3}$_EGFP or (M) EHMT1$^{V09}$_EGFP, blots probed with GFP and mCherry.

*Major concern 3.0: Result 3 (lines 182-251): Interaction between EHMT1 and viral and cellular partners. It would be preferable to separate this result from the identification of the N/C form to clarify the message by first presenting these interaction data on total extracts. RNA capture data should also be presented here. Note that there is always non-specific precipitation of N, which makes the comment about N and P capture levels (line 213) questionable.*

Response: We thank the reviewer for this suggestion. Figures have been rearranged in the revised manuscript. Identification of the form is presented in Figure 3 and the interaction data in Figure 6.

We repeated Immunoprecipitation of EHMT1 from the cytoplasmic fraction of Uninfected as well as SeV infected cells. Western blotting of the Input as well as the elute indicated no non-specific band either in the cytoplasmic fraction of uninfected cells, nuclear fraction or IgG elute (Fig R8). An association was detected only in the EHMT1 IP condition of SeV infected cells (Fig R8), indicating that the association between EHMT1 and SeV_N is indeed specific. Additionally, by transfecting cells with mCherry tagged Nucleoprotein in infected cells, we have demonstrated that EHMT1 indeed associates with the Nucleoprotein (Fig. S6B).

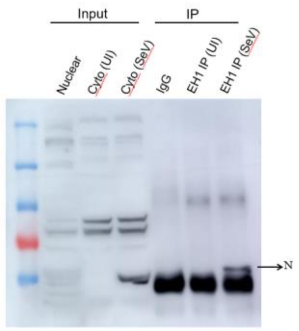


Reference Figure 8: Western blotting of elute from EHMT1 immunoprecipitated samples with respective controls. Blot was probed with antibody against Sendai virus.

***Major concern 3.1:***

*To further investigate the nature of the captured complex, the authors express mCh-N alone. The results are shown in Figure 3F. The input for the IgG control, which is necessary for the interpretation of the experiment as N tends to be immunoprecipitated specifically, is missing.*

**Response:** In this experiment, cytoplasmic fractions from mCh_N ± SeV were used for both EHMT1 IP and IgG IP. 1/10$^{th}$ of the cytoplasmic fraction was set aside to load as input. The cytoplasmic fraction was then divided into two parts, for IP and IgG. Therefore, the input was common for both EHMT1 IP and IgG control. We hope that this addresses the reviewer's concern.

***Major concern 3.2:*** *The reason for transfecting mCh-N with an infection is unclear. The combination of infection and transfection is highly inefficient (the 2 events inhibit each other). Transfected infected cells can be detected and analysed by immunofluorescence, but this is difficult to interpret by immunoprecipitation.*

**Response:** In Figure 3E (previous version), fig. S6A, Fig. 6G (revised version), we concluded that EHMT1 associated with the SeV Nucleoprotein, but the Western blot was probed with a polyclonal SeV antibody, which recognises all the 6 SeV proteins. Based on its molecular weight and abundance, we were able to conclude that the band indicated in Fig.6G, fig. S6A was the Nucleoprotein. However, to certainly state whether the band immunoprecipitated with EHMT1 was indeed the Nucleoprotein, we transfected cells with mCherry tagged Nucleoprotein and infected the cells with SeV. Majority of the cells were either transfected or infected, but a very small population of cells were both transfected and infected. However, due to the lack of specific antibodies against SeV-N, we standardised this protocol in the best possible way to perform immunoprecipitation. Western blotting of EHMT1 elute with mCherry, indicated that EHMT1 associated with Nucleoprotein in both uninfected as well as infected samples (fig. S6B), but the association was stronger in infected cells. Lack of a commercial antibody raised against the SeV Nucleoprotein led us to perform this experiment to conclusively determine this association during infection.

However, we also employed a more efficient and robust system, where co-transfection of the mCh_N along with piRFP_P resulted in majority of cells with both N+P, where IBs were formed. In this system, immunoprecipitation of EHMT1 indicated an association with mCh_N, which was stronger in co-transfection system as compared to the individual transfection of

mCh_N (Fig. 6E). Thus, our results were in alignment in both infection + transfection as well as co-transfection systems. This data is included in Fig of the revised manuscript.

***Major concern 3.3:*** *To determine the nature of the EHMT1 viral interactor, immunoprecipitation must be performed with cells transfected to express N alone, P alone and P + N.*

**Response:** We agree with the reviewer on this and performed EHMT1 Immunoprecipitation from the cytoplasmic fraction of cells transfected with N as well as N+P as suggested. Western blotting revealed that EHMT1 was Immunoprecipitated in both the conditions (Fig R9, Fig. 6E). EHMT1 IP from mCh_N transfected condition revealed an association with N, which was very weak. However, EHMT1 IP from N+P transfected condition revealed a strong interaction with the N protein, indicating that EHMT1 associates strongly with the viral Nucleoprotein only when IBs are formed, either by infection or co-transfection (Fig. 6E and G).

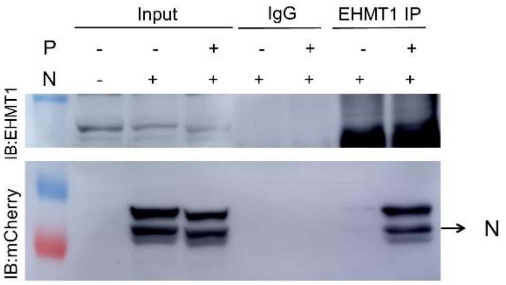


Reference Figure 9: Western blotting of EHMT1 IP from the cytoplasmic fraction of cells transfected either with mCh_N or N+P, blots probed with EHMT1 and mCherry.

***Major concern 4.0:*** *Result 4 (lines 252-303): Inhibition/KO of EHMT1 limits viral replication The qPCR protocol on which this conclusion is based needs to be detailed. It is not specified which primer is used for RT, how the authors ensure that the PCR is strand specific (or would it be better to talk about total viral RNA), whether the points are made in duplicate or triplicate, and whether there is any standardisation to cellular targets. A factor of 2 corresponds to a Ct difference that is very small and difficult to interpret.*

**Response:** For qPCR, cells were harvested in Trizol at the mentioned time points for total RNA isolation. The RNA thus isolated was then reverse transcribed to cDNA using random

hexamers and oligodT. RNAse H enzyme was then used to digest the RNA from the DNA-RNA hybrid. This cDNA was used directly to set up a qPCR. The RT-PCR primers were designed for the Sendai viral genomic RNA sequence AB039658.1; the primers span the intergenic regions between the N and P genes. The forward primer spans from 1736 to 1758 and the reverse primer spans from 1835 to 1855 (sequences of which have been provided in the manuscript); the product thus amplified indicates the expression of the genomic RNA. It is known that Sendai Viral RdRp transcribes 6 species of mRNA corresponding to the 6 genes; each gene has a start and end sequence, these mRNAs have a polyA tail and 5' cap, the primers spanning the intergenic regions will thus not be able to bind to or amplify the viral mRNA. For all the qPCR experiments, ≥3 biological replicates were used, each point on the graph represents a biological replicate. The Ct values obtained for viral gRNA were normalised with the cellular mRNA, GAPDH.

***Major concern 4.1:*** *As Sendai virus is easy to titrate, at least some of these results should be confirmed by plaque assay titration.*

**Response:** The hemolysis assay is a more commonly used and reliable assay compared to the plaque assay for quantifying Sendai viral load. The Sendai viral Hemagglutinin Neuraminidase (HN) protein is known to bind sialic acid receptors on the cell surface, facilitating viral attachment and entry into the host. When bound to RBCs, HN proteins can disrupt the cell membrane, inducing their lysis, which results in release of haemoglobin, which is proportional to the viral load, enabling us to quantify viral replication across various conditions. We thus employed this assay to assess viral replication upon inhibition of EHMT1's enzymatic activity.

BEAS-2B cells were infected with WT SeV and treated with EHMT inhibitors, BIX and UNC. The supernatant from the infected cells ± inhibitor was collected 24h p.i. and incubated with 1% RBCs on ice for attachment, followed by incubation at 37°C for lysis of RBCs. The cells were then centrifuged and supernatant containing haemoglobin from lysed RBCs were quantified; the amount of lysis being directly proportional to the viral load. From this, we observed that hemolysis was reduced by about 50-60% in EHMT inhibitor treated cells compared to the untreated infected cells (Fig R10, Fig. 4E and F). Assessing viral genomic RNA replication upon inhibition of EHMT also resulted in a reduction of about 50%. Thus, the haemolysis assay corroborated our qPCR data, further strengthening our claim that EHMT1's

enzymatic activity is essential for viral replication. This data had been included in the revised manuscript in lines 336-347.

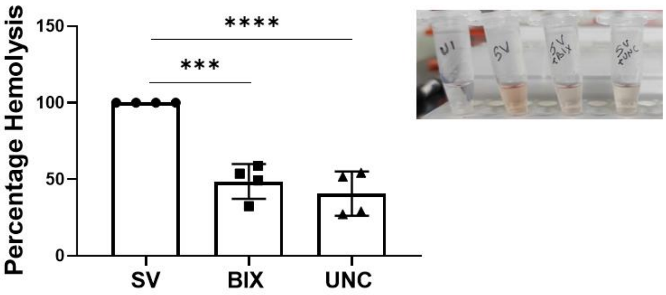


Reference figure 10: Graph representing the percentage of hemolysis upon SeV infection +/- EHMT inhibitors. Image representing the supernatant prior to measurement of absorbance.

***Major concern 5:*** *EHMTs methylate N and P. It would be interesting to see if N and P are methylated when transiently co-expressed in the absence of infection. This would confirm the link with the recruitment of EHMT1 to IBs.*

**Response:** We had performed this experiment and represented the data in fig. S6I to K of the manuscript. Co-transfection of cells with N+P and immunoprecipitation of EHMT1 from the cytoplasmic fraction revealed its association with the Nucleoprotein (Fig R11, Fig. 6E). In the co-transfection system, we couldn’t detect methylation of the Nucleoprotein (Fig R11, Fig. S6K), leading us to infer that EHMT1 methylates the Nucleoprotein upon infection. This indicated the role of additional players recruited upon infection, which could either be viral or host factors, that EHMT1 might require to methylate the N protein.

However, we demonstrated a link between IB formation and recruitment of EHMT1 by performing a time point experiment, where methylation of N was detected at the time points of complete incorporation of EHMT1 to IBs (Fig. 7I and J). Additionally, we demonstrate how EHMT inhibitors resulted in reduced methylation of N, leading to reduction in its overall levels (Fig. 6I, fig. S6G and H). This data is strongly supported by perturbation in the formation of large IBs (the population to which EHMT1 is completely recruited) upon EHMT inhibition/depletion (Fig. 8C, F, I)

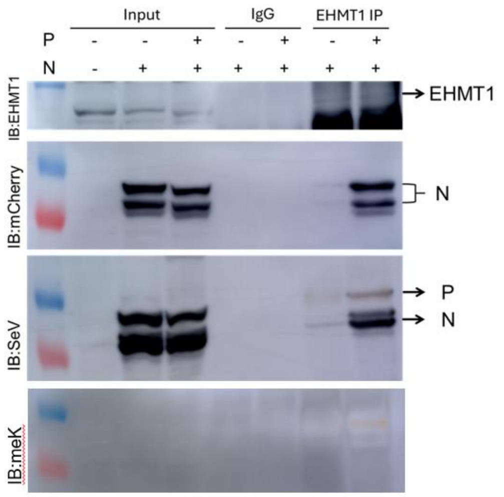


Reference figure 11: Western blotting of EHMT1 immunoprecipitated elute from the cytoplasmic fraction of either N or N+P co-transfected cells. Blots were probed with EHMT1, mCherry, SeV and meK antibodies.

***Major concern 6.0:*** *Result 6: EHMTs play a role in IB growth. Results paragraphs lines 304-308 and lines 448-501 establish the link between EHMT1 recruitment, IB size increase, replication, and methylation of viral proteins. The authors note a correlation between the size of IBs and the level of viral replication, but the results presented are insufficient to draw any conclusions.*

**Response:** Firstly, in this study, we have demonstrated that nucleo-cytoplamic forms of EHMT1 co-localize with SeV IBs (Fig. 3F and G), which are marked by proteins such as Nucleoprotein, Phosphoprotein and Large protein (RNA dependent RNA polymerase) (Fig. R12A, Fig. 2F). The localization of these three viral replication proteins to IBs itself suggests that IBs are sites of viral replication.

Next, we show that nucleo-cytoplamic form of EHMT1 is recruited to IBs by the viral Nucleo and Phosphoproteins (Fig. 2E), where EHMT1 interacts with these two viral proteins (Fig. 6G). Additionally, EHMT1 was also found to be associated with the viral genomic RNA (Fig. 6F), further strengthening our data that EHMT1 is recruited to sites of replication, where it associates with viral components.

We also demonstrate a direct correlation between the recruitment of EHMT1 progressively to the large IBs and methylation of the Nucleoprotein over the time course of infection (Fig. 7I and J). While these results establish a correlation between the size of IBs and EHMT1

recruitment as mentioned by the reviewer, our data from EHMT inhibition/depletion have demonstrated that:

Loss of EHMT1 resulted in perturbed formation of large IBs (Fig. 8C, F, I), which in turn reflected on replication (Fig. 4B and D, fig. S4B, Fig. 5B, E and G), which was reduced by about 50%. Having shown that IBs are sites of viral replication, to which EHMT1 is recruited to methylate the Nucleoprotein (Fig. 6I), loss is its activity was seen as perturbed IBs and replication (Fig. 4 and 5). While we thought that this established a link between EHMT and IB growth, to further strengthen this claim we performed following experiments

We systematically the transfected components of the viral nucleocapsid, the N, P and L proteins, along with defective viral RNA and evaluated the size of IBs. Our results demonstrated that co-expression of N & P was sufficient to recruit EHMT1 (Fig. 2E) but N, P and L factors were needed for generation of large IBs (Fig R12B, Fig. 2F, fig. S2G). Co-transfection of defective SeV RNA to N, P, L transfected cells improved large IB formation by small yet significant means (Fig. R12B). Further, addition of EHMT1 inhibitors to NPL or NPL+DI RNA inhibited the formation of large IBs (Fig. R12C, G, H and K, Fig. 4C, Fig. S4D, Fig. 8D to F), indicating EHMT1's activity in IB coalescence.

The above system helped us to conclusively state that EHMT1's enzymatic activity is essential for the coalescence/growth of IBs to form large structures in a system where viral protein concentration is controlled. We also assessed viral replication of the RNA mimic (SeV DI RNA) in mini replicon system which revealed over 80% reduction in replication (Fig. R12E, Fig. 4D) when EHMT's activity was inhibited. This reduction can be purely attributed to the perturbed formation of large IBs, given that all the other components were provided in controlled amounts across all the conditions. Therefore, we believe that this experiment helped strengthen our claim that large IBs formed by EHMT1's activity is essential for viral replication. This is now presented in detail in lines 304 - 335 of the revised manuscript.

***Major Concern 6.1:*** *They base their conclusions on the level of replication assessed by quantification of viral RNA using a non-detailed protocol. "Normalised Ct" should be defined. The normalised Ct results for the control (uninfected) are surprising (control Ct 10) and very different between the wild-type virus and the GFP virus (10 versus 25), and the T0, which is essential for interpreting the kinetics, is missing. It is necessary to detail the protocol (and explain the results if necessary), quantify the RNA at T0 and perform titrations in the lysis range to quantify the viral replication kinetics.*

**Response:** Cells were infected with either WT or EmGFP SeV and harvested at the indicated time points in Trizol for RNA extraction. Total RNA was isolated, and reverse transcribed to cDNA, which was then used for qPCR to amplify respective genes. The SeV genomic RNA was amplified by using intergenic primers designed as described in our response to Major Concern 4.0; the Ct values of which were normalised with GAPDH.

We thank you the reviewer for pointing this out. We revisited our data and realised that we had made a mistake while plotting the graph for WT SeV. We apologize for our oversight. We replotted the graph again wherein the control Ct was around 20, followed by a Ct of around 10 for 12h. This graph has been replaced with the corrected graph in the revised manuscript (Fig. 7G). In this experiment, Uninfected cells (UI) has been considered as T0, which was collected for RNA extraction at T0 (at the time point of infection of cells)

**Major concern 6.2:** For the wild-type virus, there is no difference in "Ct" between 12, 24 and 48 hours. Note that the GFP virus behaved very differently (see specific comment on this point).

Response: We agree that there is no difference between 24 and 48 hours but there is a minute, non-significant difference between 12 to 24h which suggested that replication persists until 48h around the same level. We have now modified these in our results accordingly as mentioned from line 585 to 588.

**Major concern 6.3:** The authors observed a reduction in the size of IBs when EHMT1 was inhibited, but the results did not show that this reduction in size was the cause of the reduction in viral replication. The reduction in the amount of viral proteins alone could explain this phenomenon. The fact that EHMT1 inhibitors had no effect on the pseudo-IBs formed when N and P were expressed alone could be interpreted in the same way as the authors (in favour of an effect on large IBs alone) or, on the contrary, as an argument in favour of an effect on viral synthesis. In conclusion, it is necessary to use a mini-replicon system in which the amount of viral proteins does not depend on viral synthesis.

Response: We thank the reviewer for their suggestion on the mini-replicon system. To convincingly demonstrate that EHMT1 regulates SeV replication by modulating IB size, we designed a mini replicon system as described by Calain *et al.* [18], [19], [20], Components of the viral replicon N, P and L were tagged with mCherry, far red and green fluorescent proteins

respectively, the SeV Defective Interfering (DI) RNA was commercially synthesized (Details are described in methods section). The SeV DI RNA was about $1/10^{th}$ the length of the full-length SeV gRNA, had 1400 nucleotides encompassing regions of the 5' and 3' UTR, along with a portion of the L gene. N, P, L plasmids were co-transfected in a 3:6:2 or 1:1:0.5 ratio, followed by transfection of SeV DI RNA after 12hrs. Replication of transfected SeV DI RNA was tested by qRT-PCR after 12, 16 and 24 hr wherein we observed a significant increase in replication from 12h to 16h and a progressive decrease around 24h (Fig. R12D, Fig. S4E). After validating the system, we performed the same experiment in presence or absence of EHMT1 inhibitors. Inhibition of EHMT1 activity in the mini replicon system revealed that there was a decrease in the overall size of the IBs (Fig. R12G, Fig. 8E). Noticeably, IBs of over $10\mu m^2$ size were significantly reduced (Fig. R12H and I, Fig. 8F). Analysis of the overall population of IBs did not show significant changes in small and medium IB sizes but number of large IBs were significantly reduced (Fig. R12I to K, Fig. S8E to G). These results further reiterated the significance of EHMT1 in formation of large IBs. Consistent with reduced replication in BIX/UNC treated SeV infected cells, we observed reduced replication of SeV DI RNA in the mini-replicon system as well (Fig. R12E, Fig. 4D). This reduction in replication was in response to the lack of formation of large IBs and was independent of viral replication, transcription or protein synthesis. Thus, this data further strengthened our claim that EHMT mediated regulation of large IB formation facilitates efficient replication. We have included this data in the revised manuscript (line 304 – 335, 678 – 687).

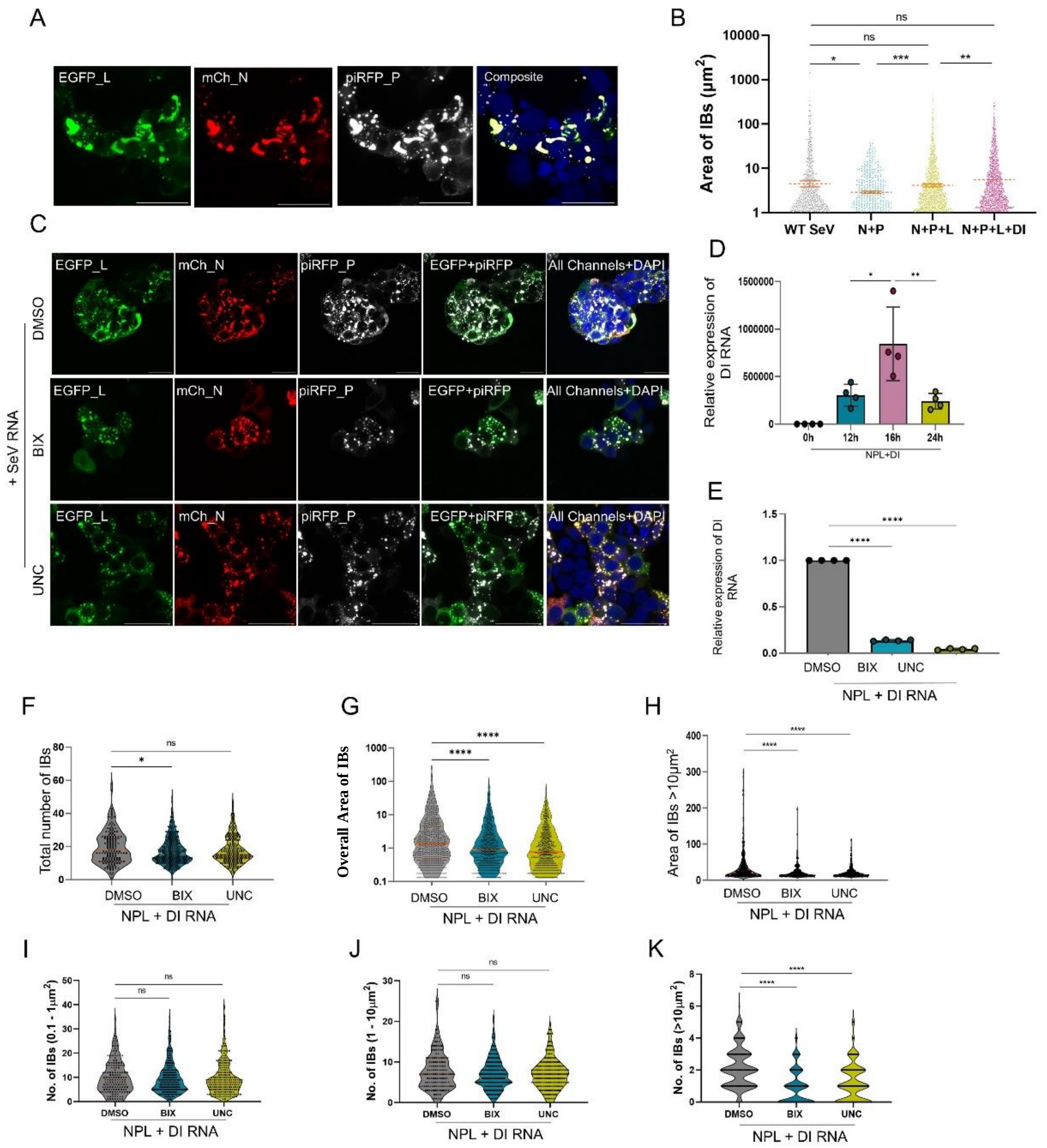


Reference figure 12: (A) Confocal microscopic images of cells co-transfected with mCh_N, piRFP_P and EGFP_L in a 3:6:2 ratio. (B) Graph representing the overall area of IBs formed upon infection or transfection of various viral components. (C) Confocal microscopic images of cells co-transfected with N, P and L treated with BIX or UNC. Nuclei are stained blue with DAPI. (D) Graph representing relative expression of SeV DI RNA at various time points post transfection. (E) Graph representing relative expression of of SeV DI RNA upon treatment with EHMT inhibitors. (F) to (K) Graphs representing the number and area of IBs formed upon EHMT inhibition, analysed from confocal microscopic images using ImageJ.

***Major concern 7:*** *Point 7: Problem with the Sendai GFP virus: This virus behaves differently from the wild-type virus: very different EHMT1 relocalisation profiles (Fig. sup1 and Fig. sup2F), very different evolution of the number and size of IBs (Fig. 5 C, D and sup Fig. 5) and different replication kinetics (Fig. 5E and F). When showing experiments based on this virus, it is necessary to indicate the conditions under which the virus was amplified, to indicate the genome structure of this virus and to compare its replication kinetics with those of the wild-type virus.*

**Response:** EmGFP Sendai virus is a proprietary vector commercialised by Thermo Fisher Scientific. From the information available on their website, we found that the Fusion gene has been deleted from the genome of this virus (Fig. R13), making it impossible for the virus to exit from the host. The Cytotune SeV kit has a polycistronic backbone, which increases the efficiency of expression of transgenes. In addition, polymerase-related genes also contain temperature sensitive mutations that facilitates faster clearance from the cells in addition to reducing cytotoxicity. Thus, the EmGFP Sendai virus has been optimised in a way by which it can enter the host and deliver transgenes; it can replicate several folds higher than the WT SeV, IBs thus formed are also much bigger in size. Our attempts to find out how the EmGFP vector has been modified by ThermoFisher Scientific has failed. So, we still do not know why and how EmGFP SeV replicates at such high magnitudes. However, both the viruses belong to the Z-strain and our intent of using both the recombinant Cytotune virus (EmGFP) and WT SeV was to demonstrate that although there are differences in the time points of infection, dynamics of IB formation and replication, EHMT1's role remains conserved regardless. Moreover, since this study was conceptualised in the beginning by using the Cytotune Virus as a vector, we continued to use this alongside the WT SeV for the rest of the study.

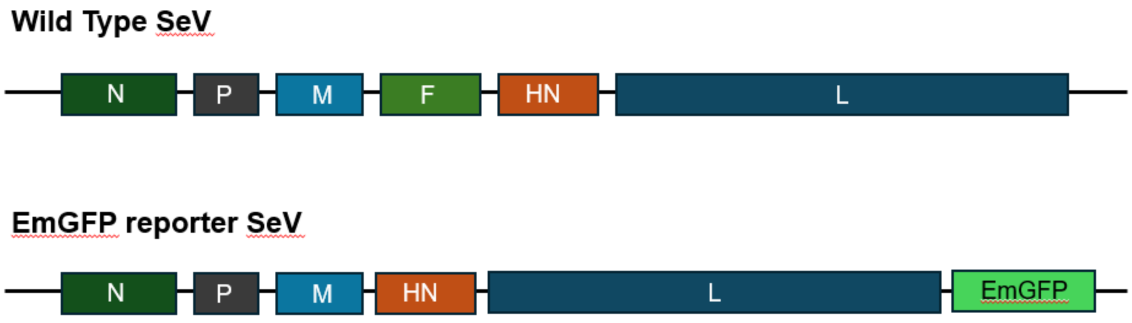


Reference figure 13: Schematic representation of SeV Wild type and EmGFP reporter SeV.

*Additional point: For all conclusions based on images without quantification, authors should indicate the number of experiments performed and the number of images taken (or cells observed).*

Response: We thank the reviewer for this suggestion. We have mentioned the numbers in the revised manuscript and included this information in a separate excel sheet. (mention where all this is included in figure legends)

*Some minor comments:*

***Minor concern 1:*** *Sup Fig3D: Explain the method used. In the uninfected case, there is as much EHMT1 in the cytoplasm and more in the nucleus than in the whole cell? on the contrary, in the infected case, there is more in the 2 compartments?*

Response: EHMT1 bands in the Uninfected and infected were normalised against the respective controls (LaminB1 for Nuclear and GAPDH for cytoplasmic). The cytoplasmic and nuclear values were further normalised against the Whole cell. However, we realise that this way of plotting could lead to improper interpretation of our results. We have therefore replaced this graph by replotting the data where the nuclear and cytoplasmic fractions are kept separate. Here, the infected values were normalised against the uninfected control (fig. S3D). We hope that this addresses the reviewer's concern.

***Minor concern 2:*** *Lines 207-209: The authors conclude that there is a reduction in EHMT1 levels in infected cells. The results do not support this conclusion. The levels appear identical in Figure 3C,*

Response: Quantification of EHMT1 bands (fig. S3D) was plotted from three independent Westerns performed on Uninfected and Infected cells at 24h p.i.. Figure 3C (previous version), however, was a time point experiment and was not considered for quantification. We also believe that Fig. 3C (previous version) and Figure 3A are redundant and removed Fig. 3C from the revised manuscript.

***Minor concern 3:*** *Figure Sup3D uses total extract as the standard for each condition, which does not allow comparison of levels between infected and uninfected cells.*

Response: We have replotted this graph, which is represented in fig. S3D of the revised manuscript.

***Minor concern 4:*** *Figure Sup3F If N means nuclear extract, why is EHMT2 abundant when it should be cytoplasmic?*

Response: EHMT2, like EHMT1 is predominantly a nuclear protein but its cytoplasmic isoform was identified in the recent years. Here, we have demonstrated EHMT2 bands in the input lanes of both Nuclear and cytoplasmic fractions. However, EHMT1 IP from the cytoplasmic fraction of SeV infected cells demonstrated an association with EHMT2 (Fig. S6E). To determine whether EHMT2 associates with SeV IBs in the cytoplasm of infected cells, we immunoprecipitated EHMT2 from the cytoplasmic fraction of infected cells and found an association with SeV N protein (Fig. S6F). These experiments confirmed the association of EHMT2.

***Minor concern 5:*** *RNA viral protein title: unclear concept. Are we talking about RNP?*

Response: By RNP, we are referring to the viral replication complex, which is the RNA bound by the N, P and L proteins.

***Minor concern 6:*** *For significance levels, do not give values, leave <0.05; <0.01; <0.001.*

Response: For statistical significance, we have mentioned only the range of p-values in the figure legends.

**Reviewer #3:**

This manuscript shows that a cellular methyltransferase, EHMT1, can be found in the cytoplasm and localizes into SeV inclusion bodies (IBs), interacting with the virus P and N proteins. Inhibition of methyltransferase activity or knockout of EHMT1 causes a reduction of SeV IBs size and levels of SeV genomic RNA, suggesting a pro-viral role for this cellular protein. However, because EHMT1 has roles in preventing expression of the antiviral interferons, this reviewer is not convinced that the reduction of the IB size and SeV genome is not at least in part due to lack of IFN repression. This major concern can be resolved by measuring IFN transcripts in controls and KO cells. If IFN is expressed to higher levels, then IFN can be blocked (using KO cells for IFN signaling elements, or neutralizing antibodies) and test for the impact of EHMT1 methyltransferase in virus replication in the absence of this confounding factor. Another major solvable concern is the overstatement and generalization of conclusions in figures that clearly show differences in only one of the conditions tested. Examples of these below.

Additional comments:

***Major concern 1:*** *Wording needs to be revise for clarity and simplicity. For example, in the abstract the authors conclude: "EHMT1 acts as proviral host factor that regulates host-pathogen interface to discern RNA viral pathogenesis". What does this means? This sentence makes no sense to me.*

**Response:** We apologize for the lack of clarity and have revised the abstract in the following way: ‘Collectively, our work elucidates a new mechanism by which cytoplasmic EHMT1 acts as a proviral host factor to regulate host-pathogen interaction.’

***Major concern 2:*** *Figure S2, D: Transfection of piRFP_P appears to cause aggregation of EHMT1, and these aggregates look very different from the ones observed during infection. However, these aggregates do not colocalize with P. What is the explanation for this observation?*

**Response:** We thank the reviewer for pointing this out, which led us to revisit our data and look at all the other images acquired while performing this experiment. We found that such aggregation wasn’t detected in the rest of the images. We also repeated this experiment to

confirm if EHMT1 forms aggregates upon P transfection and found that it does not (Fig R14, fig. S2E). Thus, we believe that the image represented in this manuscript is an outlier and we have replaced this image with another representative image (S2D). We apologize for our oversight.

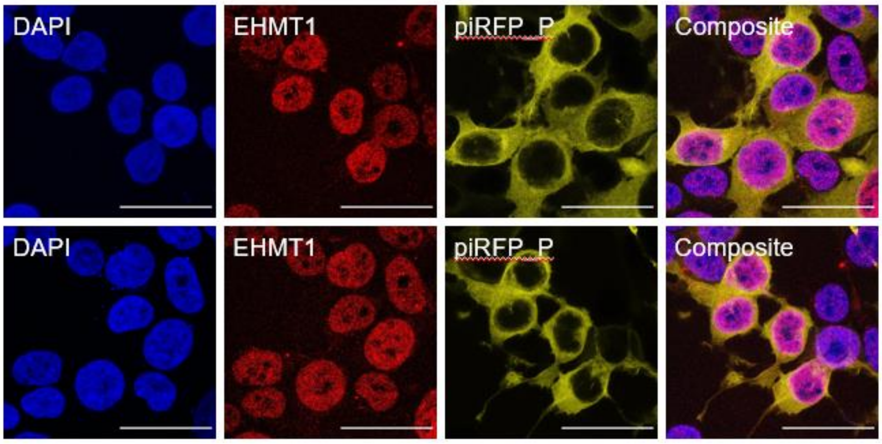


Reference figure 14: Confocal microscopic images of HEK transfected with piRFP_P (yellow), immunolabelled with EHMT1 (red), nuclei were counterstained with DAPI (blue).

***Major concern 3:*** *Lines 162-167 and Fig S2, E, F, G: Unclear what the relevance of staining for Hsp70 and how it fits into the EHMT1 story. A better explanation and rationale for using these data should be provided for clarity.*

**Response:** Hsp70, a protein folding chaperone and a stress responder, is a common host factor that is recruited into IBs formed by several viruses such as Mumps, Rabies and Respiratory Syncytial Virus [21], [22], [23], [24]. Since we were not able to find any literature describing Sendai viral Inclusion Bodies, we had to characterise them to determine whether these IBs share properties with the IBs formed by other members of the family. Thus, we looked for the expression of Hsp70 and found that it is recruited to the SeV IBs as well. Apart from its use as an IB marker, we do not intend to draw any further conclusions from this piece of data. For clarity, we have rearranged these results from line 189 - 200 in the revised manuscript.

***Major concern 4:*** *Figure 3A: Although there is clearly a band for EHMT1 in the cytoplasmic fraction, it is unclear why this signal is not observed in the images of the uninfected population of cells in Fig 1 and 2, especially considering there doesn't seem to be an upregulation of this*

*protein when comparing uninfected and infected cells. According to the methods, the same antibody was used for both techniques. A note or an explanation for this should be provided. A possibility is that this has to do with the thresholds that are being set for the imaging where in some instances are removing signal that is not background.*

**Response:** Traditionally, EHMT1 was known as a nuclear protein, whose signal is concentrated in the nuclei of cells as visualised by microscopy. In accordance with this, the threshold intensity is adjusted to capture the nuclear signal of EHMT1. However, when we set a lower threshold intensity by correcting for the background with a negative control, a faint cytoplasmic signal is observed. Here, we have acquired a few images by setting this threshold, where we see a faint cytoplasmic EHMT1 signal (Fig R15, fig. S3F), which corresponds to the bands of EHMT1 we observe in the cytoplasmic fraction upon Western blotting (Fig. 3A). The reviewer was therefore right in mentioning that the threshold was adjusted in a way that eliminates the cytoplasmic signal. Since EHMT1 wasn't known to be expressed in the cytoplasm before this study, its cytoplasmic signal used to be regarded as noise. Additionally, we have also been able to deplete this cytoplasmic form by a guide RNA targeted against the exon 3 of EHMT1 which negates any non-specificity (fig. S5D). By demonstrating the faint cytoplasmic EHMT1 signal in our microscopic images of uninfected cells and depleting the cytoplasmic protein by CRISPR/cas9, we can state that the protein is present in the cytoplasm of uninfected cells, which gets recruited upon SeV infection into viral condensates. This result is now mentioned in lines 248 - 255 of revised manuscript.

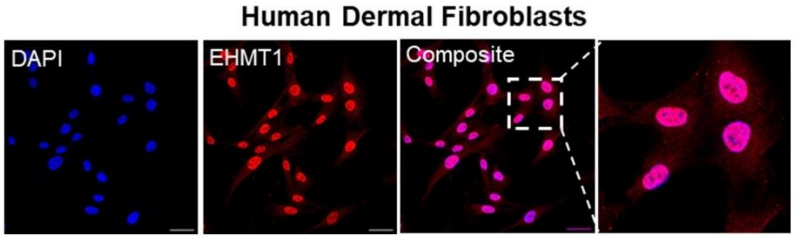


Reference figure 15: Confocal microscopic image of Human Dermal Fibroblasts immunolabelled with EHMT1 (red), nuclei counterstained with DAPI (blue).

***Major concern 5:*** *Figure 3F: Please explain why does mCherry tagged NP show two bands? And why are these bands a different size when pulling down EHMT1?*

**Response:** We do not certainly know why mCherry_NP shows two bands, but we do know that these bands are specific because they appear at the right molecular weight and are absent in untransfected cells (Fig. 6E). In addition, Western blotting of cells transfected with mCherry_NP gives us the same signal with both mCherry as well as anti-Sendai virus antibody (Fig. 6E), indicating that these bands are specific. Usually, two pools of a particular protein could arise because of post translational modifications but since we do not know how the two bands differ, we can only speculate that it could be a PTM. As such, N is known to undergo extensive posttranslational modifications.

The IP bands are slightly shifted as compared to the input bands; that is because when proteins are eluted from Dyna beads (magnetic beads used for IP), they might carry traces of the beads with them, which is hard to get rid of. Thus, the IP bands usually tend to have a slight shift in comparison with the input bands (fig. S6B). We observed this shift in the bands with both EHMT1 as well as mCherry IP (fig. S6B), indicating that it is not because of the antibody used for immunoprecipitation but rather the residual magnetic beads carried over by the elute leads to such a shift.

***Major concern 6:*** *Fig 3G: what is the bottom band that appear specifically in the IP conditions?*

**Response:** The IgG bands appear at 55KDa and the Nucleoprotein band around 58KDa. To resolve the two bands on an SDS-PAGE gel, we run it for about 5 to 6 hours until all the bands below 43KDa run out from the gel. We have noticed that changing the voltage of the run gives us two bands for the Nucleoprotein. However, IP samples run at constant voltage results in a single band as seen in Fig. 6G, Fig. S6F. We repeated EHMT2 IP and western blotted the gel to probe for SeV. Here, we have the IgG bands and a single band for SeV (Fig R16, Fig. S6F).

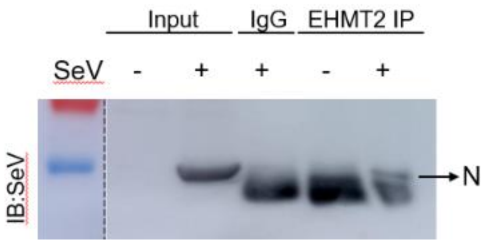


Reference figure 16: Westen blotting of EHMT2 IP from the cytoplasmic fraction of cells either infected or uninfected with SeV, blot probed with SeV.

***Major concern 7:*** *Line 262 and Figure 4C: generalizes an observation that is only found in relation to one of the drugs. Only UNC drug show differences while the BIX inhibitor shows no significant differences with the DMSO treated. This should be clearly stated and if possible, an explanation to these differences should also be provided.*

**Response:** We agree with the reviewer that BIX treated cells infected with WT SeV didn't demonstrate a significant reduction in viral replication. However, the average reduction was about 33% and due to the high error rate, it was statistically not significant. We believe that both the inhibitors are effective in terms of reducing viral replication based on our data in fig. S4B, where BIX treated cells infected with EmGFP SeV demonstrated a significant decrease in replication.

However, to verify whether BIX is effective against WT SeV replication, we repeated this experiment with the inhibitor and found that even BIX resulted in a reduction in viral replication by about 50%, which was statistically significant (Fig R17, Fig. 4B). We have now replaced the data with this graph in the revised manuscript (Fig. 4B).

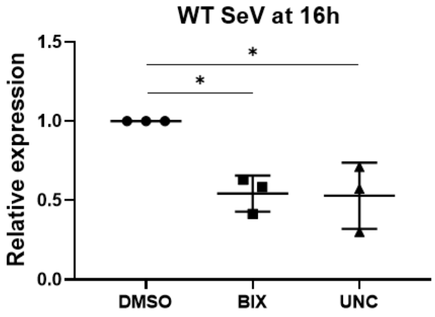


Reference figure 17: Graph demonstrating the relative expression of SeV genomic RNA upon inhibition of EHMT's enzymatic activity by treatment with the inhibitors, BIX and UNC.

***Major concern 8:*** *Figure 4K: Although complementation of the nuclear version of the EHMT1 shows no rescue of the phenotype, it does not directly demonstrate that the host response is not involved in the phenotype observed. It is assuming that both the nuclear and the nucleo-cytoplasmic have equal roles in modulating antiviral responses, which is not known given that this article just discovered the existence of this alternate version of the protein, which according to Fig 3B, also localizes in the nucleus. A more simple and direct way to demonstrate that the antiviral responses regulated by EHMT1 are not orchestrating these differences in*

*phenotype is a qPCR of IFN expression in the control vs the knockout cells. Unchanged levels of IFN in these cell lines will directly confirm the role of EHMT1 in IB size and replication is independent on its role regulating IFN.*

**Response:** We thank the reviewer for providing this valuable input to help strengthen our claim by dissecting the involvement of nuclear vs cytoplasmic EHMT1 in viral IB formation and replication. To address this, we first profiled the transcript levels of various genes belonging to the Interferon family at several time points post infection (Fig R18C and D, fig. S5E and F). Infection of cells with Sendai virus activates type 1 Interferons such as IFNβ, which facilitates clearance of virus by activation of downstream IFN stimulated genes (ISGs) like ISG20 and Ifit2 [25], [26]. BEAS-2B cells were infected with SeV and monitored for the expression of IFN related genes from 6h to Day 4 post infection. While genes like IFNβ, Ifit2 and ISG20 were early responders to SeV infection, with an increase in their expression by several folds at 6h p.i. (Fig R18C, fig. S5E), the levels of IFNβ and Ifit2 remained up to 50-fold high until Day 1 post infection (Fig R18C, fig. S5E). Similarly, IFNα levels increased 6h post infection, but its fold change in expression was lower than IFNβ; its expression however, peaked around Day1, the levels of which persisted until Day4 (Fig R18D, fig. S5F). IL8 was a late responder, whose levels increased only around Day1 and eventually peaked at Day4 post infection (Fig R18D, fig. S5F). Other genes like IRF3 and TNFα remained transcriptionally unaltered upon infection (Fig R18D, fig. S5F). Therefore, by profiling the expression of IFN related genes, we could conclude that the strain of Sendai virus we employed in this study induces an upregulation in the levels of Interferon related genes, which could in turn impart an antiviral protection to the host, aiding in the clearance of viral infection.

To dissect whether the reduction in viral replication is influenced by the cytoplasmic or nuclear EHMT1, we depleted total cellular EHMT1 by a guide RNA targeting EHMT1 via CRISPR/Cas9. These cells were then treated with an inhibitor of the IFNβ pathway, BX795 [27]. Viral infection leads to phosphorylation of IRF3 by the kinase TBK1. Phosphorylated IRF3 translocates to the nucleus and induces transcription of Type1 IFN family of genes like IFNβ, which in turn stimulates the expression of ISG20 and Ifit2. Since we observed an increase in transcription of these genes upon SeV infection (Fig R18C and D, fig. S5E and F), we used an inhibitor targeting the enzymatic activity of the kinase TBK1, BX795. Inhibition of this kinase prevents translocation of IRF3 to the nucleus and activation of genes related to the pathway. This approach allowed us to block the interferon wing controlled by the epigenetic

activity of EHMT1 while allowing the us to monitor cytoplasmic EHMT1's effect on viral replication.

Cells were transfected either with control vector containing SpCas9 encoding region (SpCas), without guide RNA or with guide RNA targeting EHMT1 (E132); 36h post transfection, cells were infected with WT SeV and simultaneously treated with BX795 (Fig. R18A and B, Fig. 5F). 24h post infection, cells were harvested for RNA isolation and qRT-PCR to assess the expression of various IFNs and SeV genomic RNA. From this panel of Interferon related genes profiled by us, we picked IFNα, IFNβ, Ifit2 and ISG20 for further experiments. While IFNα levels decreased across all conditions (Fig R18E, fig. S5G), IFNβ levels demonstrated an upregulation by about 2-fold upon infection in SpCas as well as E132 transfected conditions (Fig R18F, fig. S5H), the levels of which significantly decreased in BX795 treated cells (Fig R18F, fig. S5H). Ifit2 and ISG20 also followed a similar pattern of expression where their levels were upregulated upon infection but treatment with the inhibitor, BX795 resulted in a reduction in their levels (Fig R18G and H, fig. S5I and J). From this, we demonstrated that SeV stimulates the expression of type 1 Interferon and related genes, which were reduced by treatment with the inhibitor, BX795. In this system, we quantified SeV genomic RNA replication, where we found an increase in replication in SpCas+BX795 condition (Fig R18I, Fig. 5G), which could be attributed to inhibition of the antiviral effect imparted by IFNs, thereby allowing amplification of viral replication. However, in EH_132 transfected cells ± BX795 treatment, we observed about 50% reduction in viral replication (Fig R18I, Fig. 5G). Since there was no significant difference in viral replication in cells depleted of EHMT1, with or without treatment with BX795, we could conclude that SeV replication is not entirely influenced by Interferons, or the epigenetic activity imparted by EHMT1 on Interferon regulated genes.

Next, we tested the impact of IFN inhibition on IB composition, towards this, we transfected cells with SpCas/EH_132 + BX795 treated cells, infected with SeV were fixed and processed for Immunolabelling with SeV at 24h p.i. (Fig. 5F). The total number of IBs increased upon BX795 treatment (Fig. R18J, Fig. 8J), either with or without depletion of EHMT1 (Fig. R18J, Fig. 8J). This indicated that the inhibition of the IFN pathway favours the formation of IBs. However, the total numbers were reduced in EH_132 + BX_795 as compared with SpCas9 + BX_795 condition (Fig. R18J, Fig. 8J). The overall area of IBs was higher in SpCas9+BX795 condition when compared to SpCas9 alone (Fig. R18K, Fig. 8K), however treatment with BX975 did not lead to an increase the area in EH_132 condition (Fig. 8K). Analysis of the

large IBs greater than 10μ$m^2$ area revealed a significant increase in the numbers and area in SpCas+BX_795 condition as compared with SpCas9 (Fig. R18L, Fig. 8L and fig. S8M). The large IBs however, were significantly impacted both in EH_132 and EH_132 + BX975 conditions (Fig. R18L, Fig. 8L and fig. S8M). Further in-depth analysis identified an increase in small IBs upon BX975 treatment in SpCas9 and EH_132 (Fig. R18M, Fig. 8M) albeit at different levels, indicating that inhibition of the IFN signalling pathway promotes accumulation of small IBs. Perturbed formation of large IBs in EH_132 conditions (Fig. R18L, Fig. 8L and fig. S8M), indicated that absence of cytoplasmic EHMT1 impairs coalescence even upon inhibition of IFN signalling. Taken together, these results demonstrated that while inhibition of the antiviral IFN pathway favoured the virus by promoting the formation of small IB, coalescence of IBs to form large structures was purely governed by the enzymatic activity of $EHMT1^{N/C}$. These results are included in the revised manuscript from line 390 – 434 and 709 – 729.

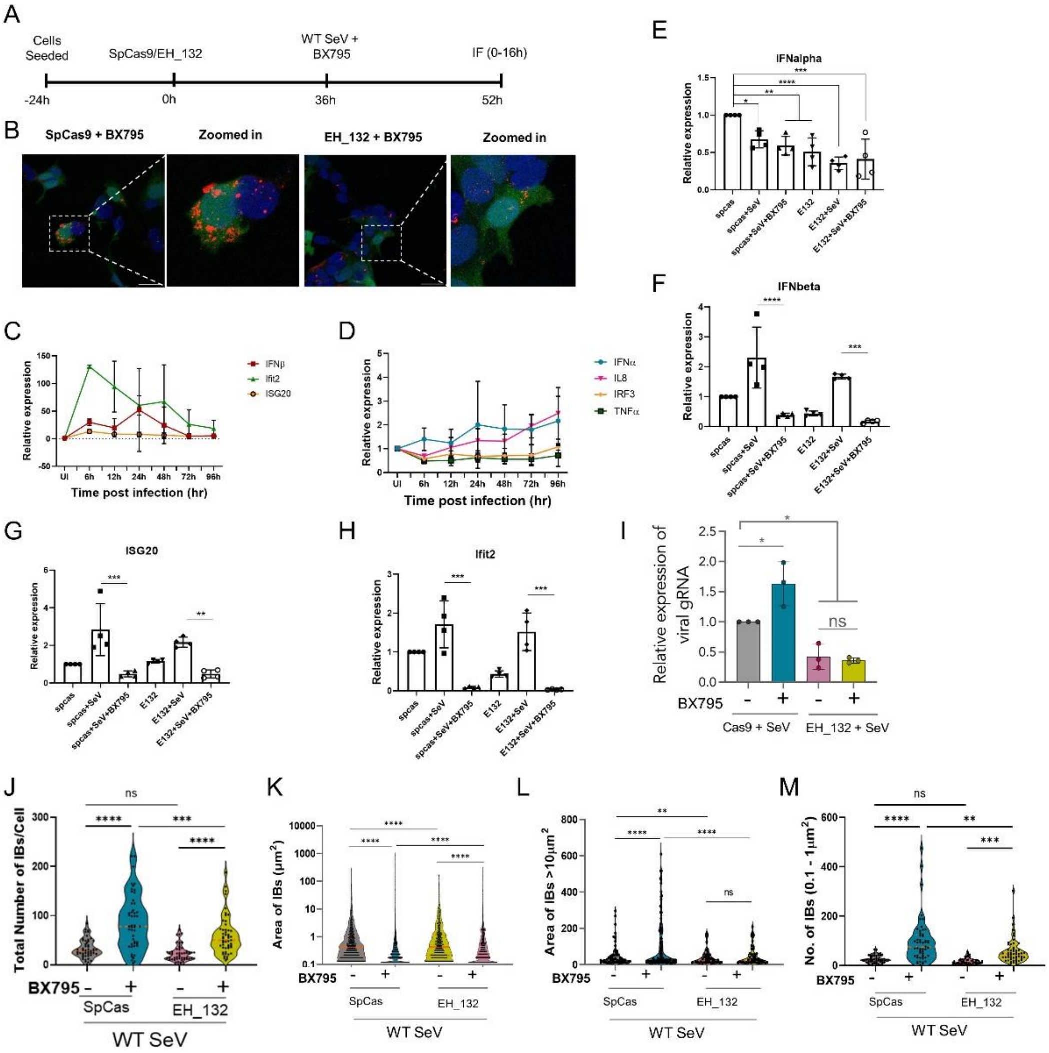

Reference figure 18: (A) Schematic representation of the experimental plan. (B) Confocal microscopic images of HEK transfected with SpCas9/EH_132, treated with BX_795 and infected with WT SeV, immunolabelled with SeV ab (red) to mark the IBs. Nuclei are counterstained blue with DAPI. (C) and (D) Relative expression of Type 1 IFN pathway genes at various time points post infection with SeV. (E) to (I) Relative expression of various IFN and related genes, SeV gRNA in cells transfected with SpCas/EH_132, ± SeV, ± BX_795. (J) to (M) Graphs representing the numbers and size of IBs of various categories, obtained by analysing confocal microscopic images by ImageJ.

***Major concern 9:*** *Figure 5C: Please explain how the boundaries of the cell were defined to calculate number of IBs per cell.*

**Response:** In the images represented below, the cells were apart from each other, so we could easily detect individual cells based on DAPI stained nuclei (Fig R19). In experiments where we had infected cells with EmGFP SeV or transfected them with pSpCas9-(bb)-2a-GFP (the plasmid where EHMT1 guide RNA was cloned), we used the GFP expression in both the cases to ensure that the right boundaries were marked (Fig R19).

To analyse the IBs formed, we used an image analysis tool, ImageJ to analyse the size and number of IBs per cell. Using the 'Freehand selections' option on this tool (Fig R19) (marked with a red box), we drew a boundary around each cell, adjusted the parameters to get the number and area of each IB formed in a cell. For analysis, either SeV or EHMT1 channel were considered individually. The tool provided us with a skeleton image with the boundaries of each IB measured (Fig R19), which enabled us to ensure that the readings are indeed coming from the IBs. Additionally, we can also click on a particular IB to determine its parameters.

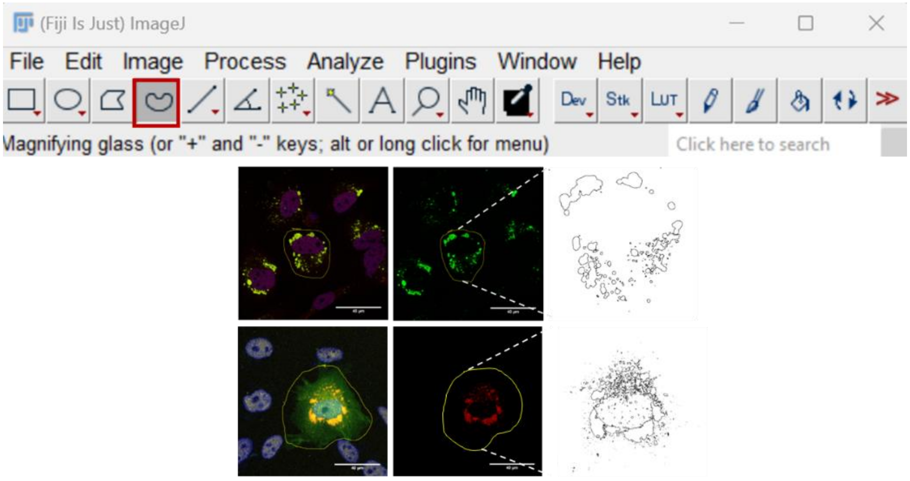


Figure R19: Image analysis tool, Image J interface (above); extracted/analysed images (below)

***Major concern 10:*** *Line 326-328 and Figure 5C: generalizes an observation that is only shown in the EmGFP infection. There is no change between the first 36 hrs in the WT infection. This should be noted and an explanation to these differences should be provided.*

**Response:** We agree with the reviewer that based on figure 5C, only EmGFP SeV demonstrates a peak in the total number of IBs at 24h, followed by a decline. While this pattern isn't true with respect to the total number of IBs formed by WT SeV, it is mainly because the total number of IBs is a collective population of all the four different size categories of IBs. Among

these, the total number of IBs is mainly dominated by the small IB (0.1 - 1μm$^2$) population, which represents over 80% of the total IBs. Additionally, for better clarity, we have separated the WT and EmGFP SeV data points from the graphs and plotted them separately (Fig. 7C and E). We thank the reviewer for this suggestion and have modified the statements accordingly in the revised manuscript (Line 562 – 566).

***Major concern 11:*** *Line 246 to 248 and Figure 5E and 5F: Unclear where the peak of replication is demonstrated. The representation of the statistics is confusing. Comparison should be made in between time points to demonstrate a "peak" of infection. There is no significant difference between times 12-48 hpi in figure 5E, demonstrating that there is not "peak" at 24 hrs.*

**Response:** [Line 346 to 348 instead] In Figure 5E, while we agree that the peak at 24h is not prominent when compared to 12h and 48h, this was the time point at which we observed maximum viral replication for WT SeV. However, due to the batch-to-batch variability among the various virus stocks used, the difference between time points was too high, which resulted in a high error bar, resulting in a different that was non-significant. Thus, for better representation in the manuscript, we plotted a graph with the normalised Ct values. The statement we tried to convey here is that IBs, being sites of viral genomic RNA replication; there was a strong correlation between the size of large IBs formed (Fig 5D and F) and the replication (Fig 5G). Similarly, figure 5H represents the replication of viral gRNA over the time course of infection, where we observed progressive increase in replication, which correlated well with an increase in the area of IBs formed over the time course of infection. Thus, both WT as well as EmGFP SeV exhibited a strong correlation between the formation of large IBs and corresponding increase in gRNA replication.

One-way ANOVA was applied to determine statistical significance in comparison to control. The significance or p-value was the same for 12, 24 and 48h indicating that there was no significant difference among these three time points. We have also replotted the graph and re-represented the statistics in the revised manuscript (Fig. 7G). Our claims have also been changed in the main manuscript text (line 585 - 588).

***Major concern 12:*** *Line 399: Should clarify that the methylation observed for the P protein is extremely minimal since the band is so faint, almost invisible.*

**Response:** Sendai virus possesses a single strand non-segmented negative sense RNA genome which encodes for 6 genes, Nucleoprotein (N), Phosphoprotein (P), Matrix (M), Fusion (F), Hemagglutinin (HN), and Large (L) in the same order. The viral RNA polymerase synthesizes each monocistronic mRNA by recognizing the start and end signal flanking each gene. While the end signal is conserved for all the genes, the start signal varies in sequence and thus strength, which in turn leads to variation in the transcription reinitiation efficiency by the polymerase. The mRNAs transcribed from the upstream genes are thus more abundant than the genes downstream of the viral genomic RNA, thus giving rise to a pool of mRNA that differ in concentration [28].

A study reported that the Sendai viral RNP consists of about 2,600 N, 300 P and 30 L protein molecules, which correlates with the study mentioned above [29]. N, being the most upstream gene is translated in abundance, followed by P and L is the least abundant of all the proteins.

In this experiment where EHMT1 was immunoprecipitated from the cytoplasmic fraction of SeV infected cells, N protein was easily detected with both SeV and meK antibodies due to its abundance. However, P being less abundant, was detected as an interaction partner but due to its levels of expression, the meK band was faint. Regardless, since we had an IgG control in which there was no band, we can state that EHMT1 associates with and methylates P.

***Minor Points***

*Line 77: missing a "the" before cytoplasmic*

*Line 136: Should say "single-strand negative-sense RNA viruses"*

*Line 158: ICC is never defined Figures 4A, 4D and 4G: These diagrams are unnecessary. The approach is straight forward and these diagrams pollute the figure.*

Response: The ICC protocol has been described in the methods section of the manuscript. We have now removed these diagrams for most of the panels.

*Line 259: Unsure what "compact" means in relation to the comparison of the IBs in the different conditions.*

Response: IBs are heterogenous structures with several numbers of IBs in infected cells. While inhibitor treated cells had smaller IBs scattered throughout the cytoplasm, control cells had larger structures. Compact refers to single large structures as opposed to scattered small structures.

*Line 298: missing a "be" after the "not"*

*Figure S5A: Graphs missing legend*

Response: We have now separated these graphs in the revised manuscript and added the legends (Fig. S7A). Since the y-axis of all the four graphs are 'Number of IBs', we have just mentioned it once.

*Figure 7: Hard to differentiate the normal NP with the methylated version. Perhaps another colour and a bigger doodle for the methylation would make it easier to see.*

Response: We have changed the colour in the revised figure.

**Response:** We thank the reviewer for suggestions on typos and missing information. We have incorporated all necessary changes to address the minor concerns in the revised manuscript.

References:


[1] T. Hoenen *et al.*, "Inclusion Bodies Are a Site of Ebolavirus Replication," *J. Virol.*, vol. 86, no. 21, pp. 11779–11788, Nov. 2012, doi: 10.1128/JVI.01525-12.

[2] Y. Zhou, J. M. Su, C. E. Samuel, and D. Ma, "Measles Virus Forms Inclusion Bodies with Properties of Liquid Organelles," *J. Virol.*, vol. 93, no. 21, pp. e00948-19, Nov. 2019, doi: 10.1128/JVI.00948-19.

[3] J. Nikolic *et al.*, "Negri bodies are viral factories with properties of liquid organelles," *Nat. Commun.*, vol. 8, no. 1, p. 58, Jul. 2017, doi: 10.1038/s41467-017-00102-9.

[4] V. Rincheval *et al.*, "Functional organization of cytoplasmic inclusion bodies in cells infected by respiratory syncytial virus," *Nat. Commun.*, vol. 8, no. 1, p. 563, Sep. 2017, doi: 10.1038/s41467-017-00655-9.

[5] S. Sarkar, S. Ganguly, N. K. Ganguly, D. P. Sarkar, and N. R. Sharma, "Chandipura Virus Forms Cytoplasmic Inclusion Bodies through Phase Separation and Proviral Association of Cellular Protein Kinase R and Stress Granule Protein TIA-1," *Viruses*, vol. 16, no. 7, p. 1027, Jun. 2024, doi: 10.3390/v16071027.

[6] J. M. Mackenzie, M. K. Jones, and P. R. Young, "Immunolocalization of the Dengue Virus Nonstructural Glycoprotein NS1 Suggests a Role in Viral RNA Replication," *Virology*, vol. 220, no. 1, pp. 232–240, Jun. 1996, doi: 10.1006/viro.1996.0307.

[7] J. Junjhon, J. G. Pennington, T. J. Edwards, R. Perera, J. Lanman, and R. J. Kuhn, "Ultrastructural Characterization and Three-Dimensional Architecture of Replication Sites in Dengue Virus-Infected Mosquito Cells," *J. Virol.*, vol. 88, no. 9, pp. 4687–4697, May 2014, doi: 10.1128/JVI.00118-14.

[8] R. E. Collins *et al.*, "The ankyrin repeats of G9a and GLP histone methyltransferases are mono- and dimethyllysine binding modules," *Nat. Struct. Mol. Biol.*, vol. 15, no. 3, pp. 245–250, Mar. 2008, doi: 10.1038/nsmb.1384.

[9] M. Tachibana *et al.*, "Histone methyltransferases G9a and GLP form heteromeric complexes and are both crucial for methylation of euchromatin at H3-K9," *Genes Dev.*, vol. 19, no. 7, pp. 815–826, Apr. 2005, doi: 10.1101/gad.1284005.

[10] X. Lahaye *et al.*, "Functional Characterization of Negri Bodies (NBs) in Rabies Virus-Infected Cells: Evidence that NBs Are Sites of Viral Transcription and Replication," *J. Virol.*, vol. 83, no. 16, pp. 7948–7958, Aug. 2009, doi: 10.1128/JVI.00554-09.

[11] M. Mavrakis *et al.*, "Rabies virus chaperone: Identification of the phosphoprotein peptide that keeps nucleoprotein soluble and free from non-specific RNA," *Virology*, vol. 349, no. 2, pp. 422–429, Jun. 2006, doi: 10.1016/j.virol.2006.01.030.

[12] Y. Zhou, J. M. Su, C. E. Samuel, and D. Ma, "Measles Virus Forms Inclusion Bodies with Properties of Liquid Organelles," *J. Virol.*, vol. 93, no. 21, pp. e00948-19, Nov. 2019, doi: 10.1128/JVI.00948-19.

[13] Y. Chang *et al.*, "Structural basis for G9a-like protein lysine methyltransferase inhibition by BIX-01294," *Nat. Struct. Mol. Biol.*, vol. 16, no. 3, pp. 312–317, Mar. 2009, doi: 10.1038/nsmb.1560.

[14] S. Kubicek *et al.*, "Reversal of H3K9me2 by a Small-Molecule Inhibitor for the G9a Histone Methyltransferase," *Mol. Cell*, vol. 25, no. 3, pp. 473–481, Feb. 2007, doi: 10.1016/j.molcel.2007.01.017.

[15] K. Katayama *et al.*, "Discovery of novel histone lysine methyltransferase G9a/GLP (EHMT2/1) inhibitors: Design, synthesis, and structure-activity relationships of 2,4-diamino-6-methylpyrimidines," *Bioorg. Med. Chem. Lett.*, vol. 30, no. 20, p. 127475, Oct. 2020, doi: 10.1016/j.bmcl.2020.127475.

[16] S. Bagai, A. Puri, R. Blumenthal, and D. P. Sarkar, "Hemagglutinin-neuraminidase enhances F protein-mediated membrane fusion of reconstituted Sendai virus envelopes with cells," *J. Virol.*, vol. 67, no. 6, pp. 3312–3318, Jun. 1993, doi: 10.1128/jvi.67.6.3312-3318.1993.

[17] *The Laboratory Rabbit, Guinea Pig, Hamster, and Other Rodents American College of Laboratory Animal Medicine*.

[18] P. Calain, J. Curran, D. Kolakofsky, and L. Roux, "Molecular cloning of natural paramyxovirus copy-back defective interfering RNAs and their expression from DNA," *Virology*, vol. 191, no. 1, pp. 62–71, Nov. 1992, doi: 10.1016/0042-6822(92)90166-M.

[19] D. Garcin, T. Pelet, P. Calain, L. Roux, J. Curran, and D. Kolakofsky, "A highly recombinogenic system for the recovery of infectious Sendai paramyxovirus from cDNA: generation of a novel copy-back nondefective interfering virus.," *EMBO J.*, vol. 14, no. 24, pp. 6087–6094, Dec. 1995, doi: 10.1002/j.1460-2075.1995.tb00299.x.

[20] L. Strahle, D. Garcin, and D. Kolakofsky, “Sendai virus defective-interfering genomes and the activation of interferon-beta,” *Virology*, vol. 351, no. 1, pp. 101–111, Jul. 2006, doi: 10.1016/j.virol.2006.03.022.

[21] H. Katoh *et al.*, “Heat Shock Protein 70 Regulates Degradation of the Mumps Virus Phosphoprotein via the Ubiquitin-Proteasome Pathway,” *J. Virol.*, vol. 89, no. 6, pp. 3188–3199, Mar. 2015, doi: 10.1128/JVI.03343-14.

[22] X. Lahaye, A. Vidy, B. Fouquet, and D. Blondel, “Hsp70 Protein Positively Regulates Rabies Virus Infection,” *J. Virol.*, vol. 86, no. 9, pp. 4743–4751, May 2012, doi: 10.1128/JVI.06501-11.

[23] A. W. Lifland, J. Jung, E. Alonas, C. Zurla, J. E. Crowe, and P. J. Santangelo, “Human Respiratory Syncytial Virus Nucleoprotein and Inclusion Bodies Antagonize the Innate Immune Response Mediated by MDA5 and MAVS,” *J. Virol.*, vol. 86, no. 15, pp. 8245–8258, Aug. 2012, doi: 10.1128/JVI.00215-12.

[24] D. C. Munday *et al.*, “Interactome Analysis of the Human Respiratory Syncytial Virus RNA Polymerase Complex Identifies Protein Chaperones as Important Cofactors That Promote L-Protein Stability and RNA Synthesis,” *J. Virol.*, vol. 89, no. 2, pp. 917–930, Jan. 2015, doi: 10.1128/JVI.01783-14.

[25] T. C. Fang *et al.*, “Histone H3 lysine 9 di-methylation as an epigenetic signature of the interferon response,” *J. Exp. Med.*, vol. 209, no. 4, pp. 661–669, Apr. 2012, doi: 10.1084/jem.20112343.

[26] C.-K. Ea, S. Hao, K. S. Yeo, and D. Baltimore, “EHMT1 Protein Binds to Nuclear Factor-κB p50 and Represses Gene Expression,” *J. Biol. Chem.*, vol. 287, no. 37, pp. 31207–31217, Sep. 2012, doi: 10.1074/jbc.M112.365601.

[27] K. Clark, L. Plater, M. Peggie, and P. Cohen, “Use of the Pharmacological Inhibitor BX795 to Study the Regulation and Physiological Roles of TBK1 and IκB Kinase ϵ,” *J. Biol. Chem.*, vol. 284, no. 21, pp. 14136–14146, May 2009, doi: 10.1074/jbc.M109.000414.

[28] A. Kato *et al.*, “Sendai Virus Gene Start Signals Are Not Equivalent in Reinitiation Capacity: Moderation at the Fusion Protein Gene,” *J. Virol.*, vol. 73, no. 11, pp. 9237–9246, Nov. 1999, doi: 10.1128/JVI.73.11.9237-9246.1999.

[29] T. Ogino, M. Kobayashi, M. Iwama, and K. Mizumoto, “Sendai Virus RNA-dependent RNA Polymerase L Protein Catalyzes Cap Methylation of Virus-specific mRNA,” *J. Biol. Chem.*, vol. 280, no. 6, pp. 4429–4435, Feb. 2005, doi: 10.1074/jbc.M411167200.
